# Sleep Apnea Syndrome (SAS) Clinical Practice Guidelines 2020

**DOI:** 10.1007/s41105-021-00353-6

**Published:** 2022-01-03

**Authors:** Tsuneto Akashiba, Yuichi Inoue, Naohisa Uchimura, Motoharu Ohi, Takatoshi Kasai, Fusae Kawana, Shigeru Sakurai, Misa Takegami, Ryo Tachikawa, Takeshi Tanigawa, Shintaro Chiba, Kazuo Chin, Satoru Tsuiki, Morio Tonogi, Hiroshi Nakamura, Takeo Nakayama, Koji Narui, Tomoko Yagi, Motoo Yamauchi, Yoshihiro Yamashiro, Masahiro Yoshida, Toru Oga, Yasuhiro Tomita, Satoshi Hamada, Kimihiko Murase, Hiroyuki Mori, Hiroo Wada, Makoto Uchiyama, Hiromasa Ogawa, Kazumichi Sato, Seiichi Nakata, Kazuo Mishima, Shin-Ichi Momomura

**Affiliations:** 1 Shiki Respiratory Clinic, Saitama, Japan; 2 Department of Somnology, Tokyo Medical University, Tokyo, Japan; 3 Department of Neuropsychiatry, Kurume University School of Medicine, Fukuoka, Japan; 4 Sleep Medical Center, Osaka Kaisei Hospital, Osaka, Japan; 5 Department of Cardiovascular Medicine, Juntendo University Graduate School of Medicine, Tokyo, Japan; 6 Department of Cardiovascular Respiratory Sleep Medicine, Juntendo University Graduate School of Medicine, Tokyo, Japan; 7 Division of Behavioral Sleep Medicine, Iwate Medical University School of Medicine, Iwate, Japan; 8 Department of Preventive Medicine and Epidemiologic Informatics, National Cerebral and Cardiovascular Center, Osaka, Japan; 9 Department of Respiratory Medicine, Kobe City Medical Center General Hospital, Hyogo, Japan; 10 Department of Public Health, Juntendo University Graduate School of Medicine, Tokyo, Japan; 11 Ota Memorial Sleep Center, Ota General Hospital, Kanagawa, Japan; 12 Department of Sleep Medicine and Respiratory Care, Division of Sleep Medicine, Nihon University of Medicine, 30-1 Oyaguchikami-cho, Itabashi-ku, Tokyo, 173-8610 Japan; 13 Department of Human Disease Genomics, Center for Genomic Medicine, Graduate School Medicine, Kyoto University, Kyoto, Japan; 14 Institute of Neuropsychiatry, Tokyo, Japan; 15 Department of Oral and Maxillofacial Surgery, Nihon University School of Dentistry, Tokyo, Japan; 16 Nakamura Clinic, Okinawa, Japan; 17 Department of Health Informatics, Kyoto University School of Public Health, Kyoto, Japan; 18 Sleep Center, Toranomon Hospital, Tokyo, Japan; 19 Department of Respiratory Medicine, Nara Medical University, Nara, Japan; 20 Uresinogaoka Samariya Bito Hospital, Okinawa, Japan; 21 Department of Hemodialysis and Surgery, Ichikawa Hospital, International University of Health and Welfare, Chiba, Japan; 22 Department of Respiratory Medicine, Kawasaki Medical School, Okayama, Japan; 23 Department of Advanced Medicine for Respiratory Failure, Graduate School of Medicine, Kyoto University, Kyoto, Japan; 24 Department of Respiratory Care and Sleep Control Medicine, Graduate School of Medicine, Kyoto University, Kyoto, Japan; 25 Department of Psychiatry, Nihon University School of Medicine, Tokyo, Japan; 26 Department of Occupational Health, Tohoku University Graduate School of Medicine, Miyagi, Japan; 27 Department of Dental and Oral Surgery, International University of Health and Welfare, Chiba, Japan; 28 Department of Otorhinolaryngology, Second Hospital, Fujita Health University School of Medicine, Aichi, Japan; 29 Department of Neuropsychiatry, Akita University Graduate School of Medicine, Akita, Japan; 30 Division of Cardiovascular Medicine, Saitama Medical Center, Jichi Medical University, Saitama, Japan

**Keywords:** Sleep apnea syndrome (SAS), Guideline, Clinical question (CQ), Continuous positive airway pressure (CPAP)

## Abstract

The prevalence of sleep-disordered breathing (SDB) is reportedly very high. Among SDBs, the incidence of obstructive sleep apnea (OSA) is higher than previously believed, with patients having moderate-to-severe OSA accounting for approximately 20% of adult males and 10% of postmenopausal women not only in Western countries but also in Eastern countries, including Japan. Since 1998, when health insurance coverage became available, the number of patients using continuous positive airway pressure (CPAP) therapy for sleep apnea has increased sharply, with the number of patients about to exceed 500,000 in Japan. Although the “Guidelines for Diagnosis and Treatment of Sleep Apnea Syndrome (SAS) in Adults” was published in 2005, a new guideline was prepared to indicate the standard medical care based on the latest trends, as supervised by and in cooperation with the Japanese Respiratory Society and the “Survey and Research on Refractory Respiratory Diseases and Pulmonary Hypertension” Group, of Ministry of Health, Labor and Welfare and other related academic societies, including the Japanese Society of Sleep Research, in addition to referring to the previous guidelines. Since sleep apnea is an interdisciplinary field covering many areas, this guideline was prepared including 36 clinical questions (CQs). In the English version, therapies and managements for SAS, which were written from CQ16 to 36, were shown. The Japanese version was published in July 2020 and permitted as well as published as one of the Medical Information Network Distribution Service (Minds) clinical practice guidelines in Japan in July 2021.

## History of medical care for sleep apnea and background of the publication of these guidelines

### History of medical care for sleep apnea syndrome

**Fig. 1:**
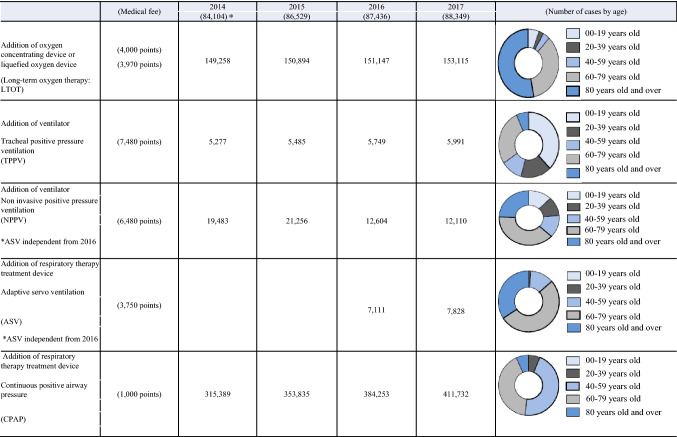
Actuals of the number of patients at-home respiratory management. Prepared by quoting materials from Ministry of Health, Labor and Welfare: Statistics by Social Medical Practice [1]. *The number of registered medical facilities

Since 1998, when health insurance coverage became available, the number of patients using continuous positive airway pressure (CPAP) therapy for sleep apnea has increased sharply, with the number of patients about to exceed 500,000 (Fig. 1) [1]. Among sleep apnea cases, the incidence of obstructive sleep apnea (OSA) is higher than previously believed. It is believed that patients with moderate-to-severe OSA account for approximately 20% of adult males and 10% of postmenopausal women [2]. Obesity is the most important factor for the occurrence of OSA. Since obesity has increased and sleep apnea-related accidents have also become problematic, the management for OSA is becoming an important social issue.

Adaptive servo ventilation (ASV), which is a type of noninvasive positive pressure ventilation (NPPV) therapy that has been developed for the treatment of Cheyne–Stokes respiration in patients with heart failure, was approved for health insurance coverage in 2016 as an item of CPAP-related machines. Therefore, it is difficult for clinicians to use CPAP or ASV properly [3]. Furthermore, the addition of remote monitoring was counted from April 2018 as telemedicine. In addition, the designated intractable disease system was initiated in Japan, with sleep apnea as an important pathological condition that needs to be distinguished from the sleep-related hypoventilation disorder of designated intractable alveolar hypoventilation syndrome.

### Background of preparing this guideline

Although the “Guidelines for Diagnosis and Treatment of Sleep Apnea Syndrome in Adults” by the “research group of sleep disordered breathing” was published previously (Medical Review Co., Ltd., 2005), a new guideline was prepared to indicate the standard medical care based on the latest trends, as supervised by and in cooperation with the Japanese Respiratory Society and the “Survey and Research on Refractory Respiratory Diseases and Pulmonary Hypertension” Group, of Ministry of Health, Labor and Welfare, and other related academic societies, in addition to referring to the previous guidelines [4, 5].

### Transition of definition and handling in these guidelines

Although respiratory disorders seen during sleep were often described as sleep-disordered breathing (SDB), it is described as sleep-related breathing disorders (SRBD) in the International Classification of Sleep Disorders, 3rd Edition: (ICSD-3). Therefore, in principle, SRBD is used instead of SDB in this guideline. However, to deepen understanding and avoid misunderstandings, they are listed together if necessary.

Sleep apnea syndrome includes obstructive sleep apnea syndrome (OSAS) or obstructive sleep apnea–hypopnea syndrome (OSAHS), as well as central sleep apnea syndrome (CSAS). OSAS or OSAHS (OSAS and OSAHS are synonymous) is common in terms of frequency, and it is often considered that SAS = OSAS or OSAHS not only in Japan, but throughout the world. In principle, this guideline clearly distinguishes between OSAS and CSAS in terms of description. Since SAS is the vocabulary often used in general clinical practice, the general name of SAS is also left as appropriate in this clinical practice guideline to avoid misleading readers and make the guideline easy to understand. Similarly, there is a document that considers OSA as OSAS, which is handled in the same manner.

Respiratory disorders during sleep are expressed in accordance with the apnea hypopnea index (AHI), and may also be evaluated in accordance with the oxygen desaturation index (ODI). The respiratory event index (REI), which is calculated by adding together the previous two disorders as the number of sleep-disordered breaths per hour of measurement, is also used for evaluation. This is sometimes described as the respiratory disturbance index (RDI) in Japan (current definition of RDI: sleep apnea per hour of sleep + hypopnea + respiratory effort-related arousal). While maintaining consistency, this guideline uses them appropriately with easy-to-understand explanations. Furthermore, in ICSD-3, it is stated in the section on diagnosis of OSAS in adults that out-of-center sleep testing (OCST) is acknowledged as portable monitoring. While OCST usually monitors multiple parameters, measuring oxygen saturation alone is sometimes called portable monitoring in Japan. Therefore, in principle, the term portable monitoring was used in this guideline. However, the phrase OCST was also used when necessary in remarks and tables.

## Objective, target, and preparation method of this guideline

### Objective

Since sleep apnea is an interdisciplinary field covering many areas [6], this guideline was prepared with the role and purpose of providing an understanding of the pathological conditions, clinical symptoms, dissemination of testing techniques, and introduction of evidence of their current effectiveness.

### Users

Since sleep apnea is managed by a medical team rather than by a physician alone, the targets are physicians and entire medical teams.

### Target patients

**Fig. 2:**
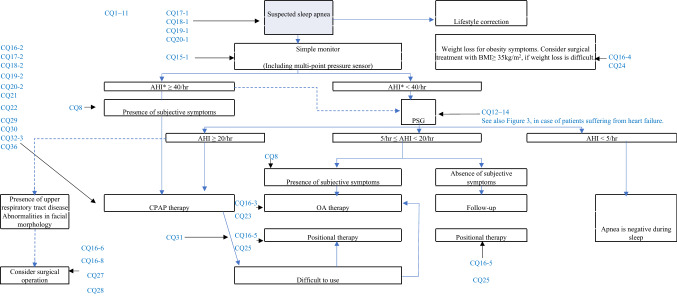
Algorithm for the diagnosis and treatment of sleep apnea, considering medical insurance treatment. *AHI includes PSG AHI, simple monitor respiratory event index (traditional Japanese respiratory disturbance index), and oxygen desaturation index. Dotted line: examination or treatment options that may be considered. (The Japanese Circulation Society, Guidelines for Diagnosis and Treatment of Cardiovascular Diseases (2008–2009 Joint Research Group Report) Guidelines for the Diagnosis and Treatment of Sleep Respiratory Disorders in the Cardiovascular Area, which was prepared by referring to < http://www.j-circ.or.jp/guideline/pdf/JCS2010. momomura.h.pdf > [4])

**Fig. 3:**
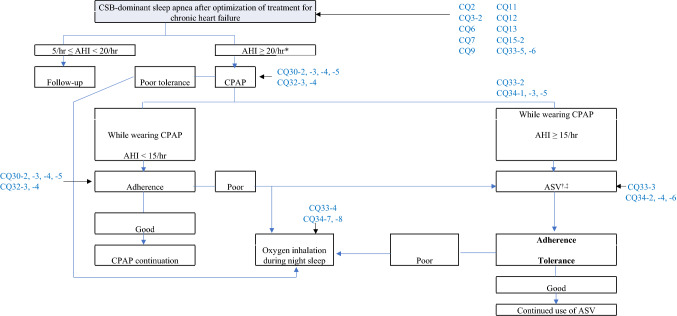
Treatment strategy for Cheyne–Stokes Breathing (CSB) associated with chronic heart failure. *≥ 15/h appears to be appropriate with regard to this AHI standard, but can be changed to ≥ 20/h taking into consideration the adaptive standard for CPAP medical insurance treatment. ^†^Attention should be paid to patients with heart failure due to decreased left ventricular contractile function (left ventricular ejection fraction ≤ 45%), which are in a stable state with central-dominant sleep apnea (statements by The Japanese Circulation Society, and The Japanese Heart Failure Society). Joint Guideline by The Japanese Circulation Society and The Japanese Heart Failure Society, for Acute/Chronic Heart Failure Clinical Practice Guidelines (2017 revised edition). ^‡^In Japan, ASV is used for severe congestion regardless of the presence or absence of sleep apnea, with ASV use for patients with heart failure approved. (Japanese Respiratory Society NPPV Guideline Preparation Committee (ed.): NPPV (Non-Invasive Positive Pressure Ventilation Therapy) Guideline, 2nd Edition, quoted and modified from p.129, 2015.)

Target patients are those suspected of having sleep apnea. Health insurance coverage was used as the standard for CPAP therapy at home. The flow charts for examination, diagnosis, and treatment are also based on the current application of health insurance in Japan (Figs. 2 and 3) [4, 5, 7, 8].

### Preparation method

Clinical questions (CQs) were prepared on the clinical features, pathophysiology, epidemiological features, laboratory procedures, and treatment of sleep apnea. Since sleep apnea is also important in relation to traffic accidents and other accidents, corresponding CQs have also been prepared. Since April 2018, the addition of remote monitoring was allowed for CPAP [3], and CQs for remote monitoring were also prepared. The CQ structure of this guideline is divided into Background Questions (BQs) and Foreground Questions (FQs), with the strength of recommendation added to FQ. In particular, in “Chapter IV. Treatment and Prognosis of SAS”, the general treatment was mentioned in FQ for both OSA and Cheyne–Stokes breathing (CSB) patients.

The level of evidence and the strength of recommendation are based on the Minds evaluation method [see the section on evidence (EBM)] [9]. The level of evidence and the strength of recommendation were mentioned every time in the FQ as well as the level of evidence in the BQ, if possible.

Upon completion, the first draft was evaluated by the Japanese Respiratory Society Guidelines Enforcement Management Committee and the Board of Directors. We further requested the Japanese Society of Sleep Research, the Japanese Circulation Society, the Oto-Rhino-Laryngological Society of Japan, Inc., the Japanese Society of Psychiatry and Neurology, the Japanese Academy of Dental Sleep Medicine, and the Japan Society for Occupational Health to provide additional external evaluation. Subsequently, the revised manuscript was published on the website of the Japanese Respiratory Society, and public comments were solicited for the revision or addition of any necessary items.

### Ingenuity to promote this guideline

A list of CQs and statements has been posted to make it easier to see, and a flowchart for diagnosis and treatment has also been posted. The CQs and answers were publicized on the website of the Japanese Respiratory Society, among others, and opinions were widely sought to gain credibility.

### Revision schedule

In principle, a revision will be made every 5 years as a guide. We pay attention to the content and trends of international guidelines, as well as revisions to the health insurance system of Japan, including cost aspects.

### Precautions for use

Sleep apnea appears frequently in various pathological conditions, and is even more frequent in patients with lifestyle-related diseases, such as hypertension and diabetes. However, it is an event which occurs during sleep, so the awareness of patients is poor. While there are various treatment methods such as CPAP, oral devices, weight loss, and surgical operations, it is difficult to set a guideline for their therapeutic effects, unlike when taking drugs. The side effects of treatment are often not clearly indicated either. Therefore, when using CPAP, it is necessary to provide patients and their families with sufficient explanations and appropriate timing to use it, in addition to paying sufficient attention to treatment policies and risk management issues.

Excessive daytime sleepiness and lack of attention may cause or induce various accidents such as traffic accidents, leading to the potential occurrence of trials and lawsuits; however, this guideline was created for use in the clinical setting, and is not intended for use in lawsuits or trials.

## Organization of the committee

To be able to garner opinions from various fields, physicians coming from fields related to sleep apnea, as well as nurses and epidemiological professionals, have kindly joined the committee.

In addition, because clinical departments where sleep apnea is frequently treated include respiratory medicine, cardiology, otolaryngology, psychiatry, dentistry, and oral surgery, we also asked the physicians of already-mentioned academic societies and specialized industrial physicians to be external evaluation committee members.

## Description concerning evidence-based medicine (EBM) [9]

(1) The level of evidence and strength of recommendation are based on the Minds evaluation method [9]. The level of evidence and strength of recommendation are stated without fail in the “Foreground Question”, with the level of evidence added if possible in the “Background Question”.

- Strength of recommendations

1. (Strong): recommended to “implement” or “not implement”.
2. 2 (Weak): propose to “implement” or “not implement”.
  No recommendation.

- Level of evidence

A (Strong): strongly confident in the estimated effectiveness.
B (Moderate): moderately confident in the estimated effectiveness.
C (Weak): confidence in the estimated effectiveness is limited.
D (Very weak): the estimated effectiveness is, for the most part, uncertain.

The literature search period subject to evaluation is as follows.

From 1966 to December 31, 2017

Papers from 1966 to December 31, 2017 were searched mainly in PubMed, the Ichu-shi Medical Journal, and the Cochrane Library. Approximately 20 papers were carefully selected for each CQ. Famous and historical textbooks and the views of the academic society were also referred to. In addition, the latest important literature has been added. Upon preparation of the manuscript, two or more members reviewed it, and the cooperators also checked for the omission of any documents to make corrections and additions.

In addition to the literature search, the latest references published during the preparation of the guideline, which were considered important, were added. The strength of recommendation was, in principle, based on papers published within the search period.

2) Selection criteria for evidence

The literature was adopted mainly for randomized controlled trials (RCTs) and observational studies in the research design. Their languages were Japanese and English. Literature on animal experiments and genetic experiments were excluded.

3) Determination of the strength of recommendation

The drafting committee held a panel meeting (for all members including proxies) to determine the strength of recommendation, and to make a decision on the strength and statement of the recommendation. During the voting regarding the strength of recommendation, those that were controversial were debated on the spot, and a re-vote was taken. As the results of the voting were almost the same, as will be described later, there was no need to use the Delphi method.

**CQ 16. OSA treatment/indications**

**CQ 16–1 What are the treatments for OSA? [BQ]**

- CPAP treatment, OA therapy, weight loss, and surgeries for nasal/pharyngeal airway patency (palatine tonsil, adenoid removal, etc.) [Evidence level: A].

**CQ 16–2 What type of OSA patients should undergo CPAP treatment? [FQ]**

- CPAP treatment is effective for OSA, with CPAP treatment recommended as the first choice for patients with strong clinical symptoms such as daytime sleepiness due to OSA, and for moderate-to-severe cases. [Strength of recommendation: 1 (Consensus rate: 100%)] [Evidence level: A].

**CQ 16–3 For what type of OSA patients is OA therapy effective? [FQ]**

- We suggest that this be performed in mild to moderate cases for which CPAP treatment is not indicated, or for cases in which CPAP cannot be used. [Strength of recommendation: 2 (Consensus rate: 100%)] [Evidence level: B].

**CQ 16–4 For what type of OSA patients is weight loss therapy effective? [FQ]**

- Weight loss therapy is recommended for obese OSA patients. [Strength of recommendation: 1 (Consensus rate: 100%)] [Evidence level: C].

**CQ 16–5 Is positional therapy effective in treating OSA patients? [FQ]**

- Some OSA patients have apnea alleviated by sleeping in a non-supine position (mainly in a lateral position). We propose that patients with mild cases, along with those who have difficulty with standard treatment such as CPAP treatment, should be instructed regarding their sleeping position, upon confirming that apnea can be reduced in the lateral position. [Strength of recommendation: 2 (Consensus rate: 100%)] [Evidence level: D].

**CQ 16–6 For what type of OSA patients is otorhinolaryngological surgery effective? [FQ]**

- If CPAP or OA cannot be used and there is an indication for otolaryngological surgery, we suggest that it be performed after fully explaining the side effects of the surgery. [Strength of recommendation: 2 (Consensus rate: 100%)] [Evidence level: C].

**CQ 16–7 For what type of OSA patients is oxygen therapy effective? [FQ]**

- Oxygen therapy may be performed on patients who cannot use CPAP or OA. [No recommendation] [Evidence level: C].

**CQ 16–8 For what type of OSA patients is maxillofacial plastic and reconstructive surgery effective? [FQ]**

- If CPAP or OA cannot be used and maxillofacial plastic and reconstructive surgery is indicated, we suggest that it be performed after fully explaining the side effects of the surgery. [Strength of recommendation: 2 (Consensus rate: 100%)] [Evidence level: C].

**Remarks**

**a. OSA treatment/indications (refer to the flowchart for indications)**

Therapeutic effects are determined by prognosis, reduction of cardiovascular risk factors, effect on complications such as hypertension, quality of life (QOL), effect on subjective symptoms such as drowsiness, and improvement of various indicators in sleep tests. OSA sleepiness improves with proper treatment. **CQ 10–2**: Strength of recommendation: 1, Evidence level: B.

For OSA patients with insomnia, prioritize treatment of OSA without using hypnotics first. **CQ 32–1**: Evidence level: C. Side effects of hypnotics include an increase in the number of events and an extension of event time in severe cases. **CQ 32–2.**

**b. CPAP treatment**

CPAP treatment has been reported to improve prognosis and many other related pathologies as follows:

A hypotensive effect is seen through CPAP treatment, with a hypotensive effect expected especially in treatment-resistant hypertension. **CQ 17–2**: Evidence level: A.

The evidence for its effects on improving glucose metabolism is insufficient. **CQ 18–2**: Evidence level: C.

The evidence for its effects on improving dyslipidemia is insufficient. **CQ 19–2**: Evidence level: C.

CPAP treatment does not reduce visceral fat within 3 months of treatment. **CQ 20–2**: Evidence level: B.

Regarding QOL, improvement can be expected for certain factors. **CQ 21–1**: Evidence level: B.

Many reports indicate that cardiovascular disorder-related parameters improve. **CQ 22–1**: Evidence level: A. Prognosis may improve if usage is maintained. **CQ 22–2**: Evidence level: B.

The side effects of CPAP treatment are described in **CQ 22–3**.

Furthermore, CPAP usage time influences its therapeutic effects on OSA patients. CQ 29–1: Evidence level: B, with improvement of daytime sleepiness having been reported with nightly use for 4 h or more. CQ 29–2: Evidence level: B, with improvements in hypertension and cardiovascular events having also been reported, with nightly use for more than 4 h. CQ 29–3: Evidence level: A.

CPAP treatment reduces the risk of road accidents. CQ 35–2: Evidence level: B.

CPAP treatment should be continued, because OSA recurs due to interruption of CPAP treatment. CQ 31–1: Evidence level: A.

Regarding adherence improvement, there is no difference in CPAP adherence between fixed pressure and auto CPAP, as long as the appropriate pressure is set. CQ 30–1: Evidence level: C. No improvement has been found in CPAP adherence due to the pressure relief function. CQ 30–2: Evidence level: C. Choosing the best mask for the patient may improve CPAP adherence. CQ 30–3: Evidence level: C. Adherence may be improved using a humidifier or nasal drops in case of nasal congestion. CQ 30–4: Evidence level: C. Supportive interventions, educational interventions, behavioral therapies, etc. also improve CPAP adherence. CQ 30–5: Evidence level: C. The combined use of CPAP treatment and hypnotics has been proposed if there is insomnia even with the CPAP treatment under appropriate settings, because it is considered effective in improving CPAP adherence. CQ 32–3: Strength of recommendation: 2, Evidence level: C.

Remote monitoring can be expected to improve CPAP adherence as well as reduce medical costs and labor on the part of medical staff. CQ 36–1: Evidence level: C.

**c. OA therapy**

QOL can be expected to improve in certain factors using OA. **CQ 23–1**: Evidence level: B, with some cardiovascular risk factors also improving. **CQ 23–2**: Evidence level: C. Side effects are described in **CQ 23–3**. Although OA is less effective than CPAP, it is considered to have a long usage time. It is also necessary to confirm the effect of OA and conduct regular follow-up. OA is also effective against snoring but is not covered by insurance.

**d. Weight loss and lifestyle guidance**

Apnea can be reduced for obese patients by providing them with standard treatment for OSA, including weight loss guidance. **CQ 24–1**: Evidence level: C. Weight loss may improve quality of life and cardiovascular risk factors. **CQ 24–2**: Evidence level: D. **CQ 24–3**: Evidence level: C.

Weight loss surgery has different effects depending on the surgical procedure, and there are management problems such as weight gain following surgery. In addition to weight loss guidance, drinking alcohol may worsen when accompanied by obesity (**CQ 3**: Refer to the Remarks); thus, a guidance on alcohol abstinence will be provided.

Regarding smoking, 20 cigarettes a day is considered to be equivalent to a prognosis of mild to moderate OSA; thus, smoking should be stopped [10]. Smoking is associated with severe OSA [11] and has been reported to be exacerbated by inflammation of the upper respiratory tract caused by smoking [12]; however, some reports refute this [13]. It has also been reported that women are at an increased risk of cardiovascular disease [14]. There are currently no reports of whether smoking cessation has improved OSA.

**e. Postural therapy**

The devices used for postural therapy have not been standardized. For patients with mild cases or who have difficulty with standard treatment such as CPAP, instructions are provided regarding their sleeping position, upon confirming that apnea is reduced in the lateral position. **CQ 25–1**: Evidence level: D.

**f. Otorhinolaryngological surgery**

Otorhinolaryngological surgery can be expected to improve QOL in OSA patients under certain circumstances, **CQ 27–1**: Evidence level: C, and AHI may improve following surgery, which may improve cardiovascular disorders. **CQ 27–2**: Evidence level: D. Side effects are described in **CQ 27–3**.

In the event that CPAP and OA cannot be used and there are pathological conditions indicated for surgery, such as anatomical abnormalities that are expected to worse AHI, and furthermore, if the effects of surgery are considered to outweigh the side effects of surgery, the side effects should be fully explained to the patient before performing the surgery.

**g. Oxygen therapy**

For cases in which CPAP and OA cannot be used, oxygen therapy is possible if desired; however, it is not covered by insurance. That said, in the event a patient with chronic heart failure has been diagnosed by a physician as having a NYHA functional classification of III or higher, Cheyne–Stokes respiration during sleep is observed, and polysomnography has confirmed an AHI of 20 or higher, oxygen therapy is covered by insurance for such patients.

The rationale for improving quality of life with oxygen therapy is unclear. **CQ 26–1**: Evidence level: D. Whether oxygen therapy improves nocturnal hypertension in OSA patients is controversial. **CQ 26–2**: Evidence level: C. The rationale for improving diabetes is unclear. **CQ 26–3**: Evidence level: D. There is little evidence that oxygen therapy is useful in controlling the onset of cardiovascular disease. **CQ 26–4**: Evidence level: C. As a side effect, it may prolong the duration of the apnea event, causing hypercapnia. **CQ 26–5**.

**h. Maxillofacial plastic and reconstructive surgery**

Improved AHI and QOL due to morphological changes in the face are expected for cases in which CPAP and OA cannot be used. **CQ 28–1**: Evidence level: D. Surgery may improve AHI and cardiovascular disease. **CQ 28–2**: Evidence level: D. Side effects are described in **CQ 28–3**. If there is a condition for which surgery is indicated and if its positive effects are considered to outweigh its risks, the latter should be fully explained to the patient before performing the surgery.

**CQ 17. OSA and hypertension**

**CQ 17–1 Does OSA cause high blood pressure? [BQ]**

- OSA is one of the main causes of secondary hypertension; thus, special attention should be paid to complications of OSA in treatment-resistant hypertension, as well as in nocturnal and early-morning hypertension. [Evidence level: A].

**CQ 17–2 Does CPAP treatment improve hypertension in OSA patients? [BQ]**

- CPAP treatment lowers the blood pressure of OSA patients and can be expected to have a hypotensive effect, in addition to weight loss and antihypertensive drugs. [Evidence level: A].

**Remarks**

**a. Relationship between OSA and hypertension**

OSA and hypertension have a high rate of complications, with hypertension occurring in approximately 50% of OSA patients and OSA occurring in approximately 30% of hypertensive patients. A prospective cohort study suggested that OSA and hypertension are not just comorbidities, with untreated OSA causing hypertension while adherent CPAP treatment possibly able to prevent the onset of hypertension [15]. On the other hand, an RCT of asymptomatic OSA patients found no difference in the risk of developing hypertension, depending on the presence or absence of CPAP treatment; however, it also indicated that the risk of developing hypertension may be reduced in the subgroup that used CPAP treatment 4 h per day or more [16]. These results suggest that OSA phenotypes and adherence to CPAP treatment may affect one’s risk of developing hypertension.

**b. Characteristics of hypertension associated with OSA**

The hypertension associated with OSA is characterized by non-dipping type nocturnal and/or early-morning hypertension [17]. OSA is considered to be the most important cause of secondary hypertension and was diagnosed in approximately 70% of treatment-resistant hypertension patients, whose blood pressure do not normalize even when three or more different classes of antihypertensive drugs are used [18].

**c. Antihypertensive effect of CPAP treatment**

While the treatment of hypertension is important to control death or reduced quality of life due to cardiovascular disease [19], CPAP has been shown to reduce blood pressure in OSA patients. A meta-analysis of 29 RCTs found that CPAP decreased systolic blood pressure by 2.6 ± 0.6 mmHg (95% CI 1.4–3.7) and diastolic blood pressure by 2.0 ± 0.4 mmHg (95% CI 1.2–3.8). Studies using a 24-h sphygmomanometer also indicated that CPAP lowered blood pressure during both the daytime and nighttime [20]. Similar results were obtained in other meta-analyses, demonstrating that OSA is a direct cause of hypertension [21]. Factors associated with a more promising antihypertensive effect of CPAP include poor baseline blood pressure control, severe OSA, strong sleepiness, and good CPAP treatment adherence [20, 22]. A meta-analysis of four RCTs in OSA patients with treatment-resistant hypertension also exhibited that CPAP reduced systolic blood pressure by 6.7 mmHg (95% CI 3.5–10.0) and diastolic blood pressure by 5.9 mmHg (95% CI 2.5–9.4) [23].

**d. Antihypertensive effect by combining CPAP treatment with other treatments**

In an RCT comparing the three treatment groups (CPAP alone, weight loss alone, and CPAP plus weight loss) in patients with moderate or severe OSA who are obese with a BMI of 30 kg/m^2^ or higher, a significant decrease was observed in systolic blood pressure in the combination treatment group compared with the CPAP alone group or the weight loss alone group (− 14.1 mmHg, − 3.0 mmHg and − 6.8 mmHg, respectively) [24]. These results indicate that weight loss is an essential treatment strategy for obese OSA patients, and at the same time, the combined use of CPAP provides an additional antihypertensive effect.

Although the hypotensive effect of CPAP was less than half that of antihypertensive drugs, CPAP has been shown to have an additional antihypertensive effect [25, 26]. The antihypertensive effect of CPAP may be used as an adjunct to antihypertensive drugs, since treating the potential cause of secondary hypertension is essential. As mentioned above, the antihypertensive effect of CPAP can be expected in treatment-resistant hypertension, even when three or more antihypertensive drugs are used.

**CQ 18. OSA and diabetes**

**CQ 18–1 Is OSA a risk factor for developing type 2 diabetes? [BQ]**

- OSA is likely to be an independent risk factor for the development of type 2 diabetes. [Evidence level: B].

**CQ 18–2 Does CPAP treatment improve glycemic control in OSA patients? [BQ]**

- The evidence that CPAP treatment can improve glucose metabolism is insufficient. [Evidence level: C].

**Remarks**

**a. Prevalence of OSA in patients with type 2 diabetes**

OSA and type 2 diabetes share common risk factors, such as obesity and aging, and are more likely to complicate each other. The prevalence of OSA (AHI ≥ 5) in type 2 diabetic patients was 65%, with 26% having moderate (AHI ≥ 15) or higher OSA [27]. Another report also found that 77% of OSAs have AHI ≥ 5, while 38% have moderate or higher OSA [28].

**b. OSA and insulin resistance**

Although the cross-sectional analysis suggests that OSA is a risk factor for insulin resistance, it is insufficient to prove a direct causal relationship given that effects of confounding factors, such as visceral fat, cannot be ruled out [29]. However, a case–control study limited to non-obese young healthy men found an association between OSA and insulin resistance, providing compelling evidence that OSA increases insulin resistance independently of age and obesity [30]. An 11-year cohort study of non-diabetic male patients also indicated that AHI and ODI are independent risk factors for insulin resistance [31].

**c. OSA and risk of developing type 2 diabetes**

The above-mentioned cohort study showed that the risk of developing type 2 diabetes in OSA patients (4% ODI ≥ 5) was also increased even after adjusting for confounding factors [31]. OSA was also an independent risk factor for developing type 2 diabetes in a large retrospective cohort [32]. A meta-analysis of prospective studies also reported that moderate-to-severe OSA increased the risk of developing type 2 diabetes [33]. Considering the relationship between OSA and insulin resistance, OSA is likely to be a risk factor for developing type 2 diabetes independent of obesity. Intermittent hypoxia associated with OSA has also been shown to be a risk for developing diabetic complications, especially peripheral neuropathy and ophthalmopathy [27].

**d. Effect of CPAP on abnormal glucose metabolism**

RCTs investigating the effect of CPAP on insulin resistance (or insulin sensitivity) showed mixed results: some studies found improved insulin resistance [34–37], while others did not [24, 38–41]. These results do not necessarily contradict the notion that OSA causes insulin resistance, because it is possible that insulin resistance is more strongly regulated by other factors (obesity and dietary/exercise habits) than simple correction of OSA. In addition, treatment effects of CPAP can potentially differ depending on the duration of CPAP treatment, adherence, and patient background.

The therapeutic effect of CPAP on glycemic control in OSA patients with diabetes or borderline diabetes is also unclear. While recent RCTs showed improved insulin resistance and glycemic control by setting rigorous treatment adherence and a relatively long treatment period [34, 37], other RCTs have failed to show improvements in these outcomes [36, 41, 42]. These mixed results may also be explained by potential confounders, including severity of OSA, changes in body weight, lifestyle modifications, and treatment adherence. Thus, the use of CPAP alone cannot be expected to have a clinically significant effect on glycemic control without surpassing other major diabetogenic factors.

**CQ 19. OSA and dyslipidemia**

**CQ 19–1 Is OSA a risk factor for dyslipidemia? [BQ]**

- The evidence that OSA is an independent risk factor for dyslipidemia is insufficient. [Evidence level: C].

**CQ 19–2 Does CPAP treatment improve dyslipidemia in OSA patients? [BQ]**

- The evidence that CPAP treatment can improve dyslipidemia is insufficient. [Evidence level: C].

**Remarks**

**a. OSA and dyslipidemia**

Animal studies have shown that intermittent hypoxia induces hepatic lipid production and causes dyslipidemia [43]. Cross-sectional studies have indicated an association between OSA and dyslipidemia independent of age and BMI [44, 45]. However, lipids were not the primary outcome in many of these studies, and visceral fat as a critical confounding factor was not taken into consideration. Overall, these cross-sectional studies do not establish a direct causal relationship between OSA and dyslipidemia.

**b. Effects of CPAP treatment on lipids**

The results of studies examining changes in lipids following CPAP treatment are inconsistent, as described below. Similar to glucose metabolism, obesity and lifestyle may act as more potent risk factors. In addition, duration of studies, adherence of CPAP, and patient background are potential confounders. Overall, the evidence that CPAP treatment improves dyslipidemia are insufficient.

**(1) Studies favoring therapeutic effects of CPAP on dyslipidemia**

An RCT of 220 OSA patients found that 1-month CPAP treatment reduced total cholesterol levels by 10.8 mg/dL [46]. However, there was no significant difference from the control group (− 2.7 mg/dL). In addition, the blood sampling time was not limited to fasting. A crossover comparative study of 29 patients with moderate/severe OSA found that 2-month CPAP treatment suppressed postprandial triglyceride (TG) elevation and decreased fasting total cholesterol and high-density lipoprotein-cholesterol (HDL) levels [47].

**(2) Studies against the therapeutic effects of CPAP on dyslipidemia**

A crossover comparative study of 34 male OSA patients found that 6 weeks of CPAP treatment did not change serum lipids. Results were similar to a sub-analysis limited to patients with good CPAP adherence [38]. A crossover comparative study of 41 OSA patient found that total cholesterol, HDL, and low-density lipoprotein (LDL) levels did not change after 2-week CPAP treatment, while TG levels were significantly elevated in the CPAP treatment group [48]. An RCT of 391 minimally symptomatic OSA patients showed no significant change in total cholesterol levels after 6 months of CPAP treatment [49]. An RCT comparing the therapeutic effects of CPAP alone vs. weight loss alone vs. CPAP/weight loss combination on 181 patients with moderate or severe OSA found that dyslipidemia was improved only in the groups including weight loss intervention, with no significant changes in TG, LDL, or HDL levels observed in the CPAP alone group [24]. A prospective cohort study of 613 patients with moderate-to-severe OSA, which compared 199 patients with good CPAP treatment adherence after adjusting for property scores and 118 without CPAP, found no difference in the amount of change in cholesterol, LDL, HDL, and TG levels between the two groups 2 years after CPAP treatment [50].

**CQ 20. OSA and visceral fat**

**CQ 20–1 Is visceral fat obesity a risk factor for OSA? [BQ]**

- Visceral fat obesity is an important factor in OSA. [Evidence level: A].

**CQ 20–2 Does CPAP treatment reduce visceral fat mass in OSA patients? [BQ]**

- CPAP treatment within 3 months does not reduce visceral fat mass in OSA patients. [Evidence level: B].

**Remarks**

**a. OSA and visceral obesity**

While obesity is an important risk factor for OSA, visceral fat obesity (abdominal obesity) increases the risk of OSA [51, 52]. The reasons why visceral fat obesity is more closely associated with OSA than subcutaneous fat obesity are: (1) abdominal obesity frequently accompanies fat accumulation around the upper airway, which anatomically narrows the upper respiratory tract [53]; (2) central adiposity leads to reductions in lung volume and loss of caudal traction on the upper airway, making it easier for the upper airway to collapse [54]; and (3) decreased functional residual capacity makes hypoxemia during apnea more severe. The influence of abdominal obesity on OSA is stronger in men [55, 56].

Metabolic syndrome resulting from visceral obesity and OSA frequently complicate each other. Approximately half of men with moderate OSA have metabolic syndrome, while nearly half of men with metabolic syndrome have moderate or severe OSA [57–59]. OSA and visceral obesity are independent risk factors for hypertension and insulin resistance through systemic inflammation and sympathetic activation, thus increasing the risk of cardiovascular disease in an additive manner [60].

**b. Effect of OSA treatment on visceral obesity**

CPAP treatment did not reduce visceral fat mass in randomized controlled trials [38, 40, 55, 61]. Rather, a meta-analysis of randomized trials demonstrated modest weight gain during the first few months following CPAP [62]. Randomized controlled trials have also indicated that CPAP treatment alone does not improve metabolic syndrome [38, 63, 64].

**CQ 21. OSA and QOL**

**CQ 21–1 Does CPAP treatment improve QOL in OSA patients? [BQ]**

- CPAP treatment can be expected to improve the QOL of OSA patients in certain aspects. [Evidence level: B].

**Remarks**

It is believed that OSA patients may have reduced QOL mainly due to daytime sleepiness. Since QOL is inherently a comprehensive and multifaceted index that includes sleep quality itself, it is difficult to determine whether QOL is decreased by OSA alone. It can be said that there is consensus on the improvement of subjective sleepiness when OSA is treated with CPAP [65].

Many research papers using SF-36 (36-Item Short Form Health Survey), which is considered the gold standard, have been reported as an index of QOL. SF-36 consists of eight subscales, and is roughly divided into physical factors and mental factors. According to a systematic review; that is, report 2 reported in 2017, CPAP therapy was reported to significantly improve QOL compared to the control for physical factors [66, 67], while report 1 reported that it improved mental factors significantly [66], although other reports report that there was no significant difference [67]. Similar meta-analyses in the past also state that the QOL, as evaluated by SF-36, was improved by CPAP [68–70]; however, these were mixed with other QOL indicators or deal with only some sub-indexes of SF-36, which is problematic.

On the other hand, from the viewpoint of mental factors, many research papers evaluating indicators of anxiety and depression have been reported. Although two meta-analyses both concluded that CPAP can improve anxiety and depression [68, 69], it has been reported that the indicators used vary, and that the effect is high when targeting people having a strong tendency to be depressed; therefore, it is not wise to generalize. It should also be noted that these psychological subjective assessments are subject to therapeutic intervention, with contact with medical staff itself tending to have a positive effect.

QOL indexes other than SF-36 include the SAQLI (Sleep Apnea Quality of Life Index) and FOSQ (Functional Outcomes of Sleep Questionnaire), which specialize in sleep quality. According to a meta-analysis that combines these, CPAP therapy significantly improves QOL compared to the control group, a result which is not affected by the presence or absence of subjective sleepiness indicated by the Epworth Sleepiness Scale (ESS) [67].

It is difficult to determine if there is improvement of QOL based on evidence because the conclusion changes depending on what is used as an index. However, improvement of QOL that cannot be explained by sleepiness alone, as an evaluation of sleep, is expected, as well as physical performance during the day regardless of sleep, which may lead to the elimination of adverse mental effects such as anxiety and depressed mood.

We believe that CPAP therapy can be proposed to improve QOL with moderate confidence based on evidence from meta-analyses.

**CQ 22. CPAP treatment for OSA**

**CQ 22–1 Does CPAP treatment improve cardiovascular parameters in OSA patients? [BQ]**

- CPAP treatment improves cardiovascular parameters in OSA. [Evidence level: A].

**CQ 22–2 Does CPAP treatment improve the prognosis of OSA patients? [BQ]**

- CPAP treatment may improve the prognosis of OSA (such as preventing cardiovascular events), as long as its usage is maintained. [Evidence level: B].

**CQ 22–3 What are the side effects of CPAP treatment? [BQ]**

- There is discomfort due to the interface, dryness, as well as skin and eye discomfort. [Evidence level: –].

**Remarks**

OSA may be associated with the development of cardiovascular disease through an exaggerated negative intrathoracic pressure during respiratory events, repetitive hypoxia and hypercapnia, enhanced sympathetic nerve activity due to frequent arousals, and vascular endothelial dysfunction as well as arteriosclerosis in association with increased oxidative stress and the inflammatory response [71–75]. More advanced arteriosclerosis is observed in severe OSA [71–75], and they have a high risk of developing fatal and non-fatal cardiovascular diseases [76]. However, treatment for OSA may improve arteriosclerosis and cardiovascular outcomes [71–76]. A recent trial, wherein patients with non-hypertensive OSA were randomly assigned to groups with and without CPAP, has indicated that despite no significant difference between the two groups, a subgroup analysis revealed that compliant patients with CPAP had a significantly lower incidence of cardiovascular events [16]. Although the effects of CPAP in OSA patients with cardiovascular disease have been reported in observational studies, in a randomized trial, named the SAVE trial, enrolling OSA patients with a history of cardiovascular or cerebrovascular disease led to no significant difference in the primary endpoint [77]. However, in a matched analysis comparing compliant patients and control group, CPAP treatment was effective with regards to cerebrovascular accidents. The RICCADSA trial, which enrolled OSA patients who had undergone percutaneous and surgical coronary intervention, did not indicate a significant difference in the primary endpoint; however, compliant patients had improved prognosis [78]. The side effects of CPAP on OSA are minor, including interface discomfort, dryness, as well as skin and eye discomfort [67].

**CQ 23. OA therapy for OSA**

**CQ 23–1 Does OA therapy improve QOL in OSA patients? [BQ]**

- OA therapy can be expected to improve the QOL of OSA patients in certain aspects. [Evidence level: B].

**CQ 23–2 Does OA therapy improve cardiovascular disease risk factors in OSA patients? [BQ]**

- OA therapy improves some cardiovascular risk factors in OSA patients. [Evidence level: C].

**CQ 23–3 What are the side effects of OA therapy? [BQ]**

- Short-term side effects associated with OA use include hypersalivation (or decreased saliva), pain and discomfort in the teeth and gums, occlusal abnormalities when waking up, and discomfort in the jaw muscles as well as temporomandibular joints. In general, these symptoms disappear over time. Long-term side effects include tooth movement and associated occlusal abnormalities, which are irreversible. [Evidence level: –].

**Remarks**

**a. Relationship between OA and QOL**

Oral appliances (OA) are classified into two types, mandibular advancement devices and tongue retaining devices, with OA generally referring to the former. This CQ refers to the former.

OA improves health-related QOL in OSA patients evaluated using SF-36 as an index. OA improved the QOL summary score of the mental aspect in SF-36 by 2.4 points (95% CI 0.0–4.9, *p* = 0.053) and the physical QOL summary score by 1.5 points (95% CI − 0.2 to 3.2, *p* = 0.076) compared to the inactive control [66]. On the other hand, CPAP improved the mental QOL summary score and the physical QOL summary score by 1.7 points (95% CI 0.1–3.2, *p* = 0.036) and 1.7 points (95% CI 0.5–2.9, *p* = 0.005), respectively, against the inactive control. Furthermore, a comparison of CPAP and OA indicated a difference in the QOL summary score for mental aspects of − 0.8 points (95% CI − 3.4 to 1.9, *p* = 0.57) and in the QOL summary score for physical aspects of 0.2 points (95% CI − 1.7 to 2.1, *p* = 0.84). Since OA is usually used in patients with relatively mild OSA, baseline OSA severity may affect the therapeutic effects of OA and CPAP. However, the severity and sleepiness of OSA patients at baseline were similar with these two treatments. Comprehensively considering the above, OA improves QOL as much as CPAP compared to the inactive control, as reported in other meta-analyses [21, 65, 67, 79, 80].

**b. OA treatment and cardiovascular disease**

Very few RCTs have seen the effects of OA in OSA patients on cardiovascular risk-related biomarkers (NT-proBNP, inflammatory markers, oxidative stress parameters, etc.), vascular endothelial function, and arteriosclerosis [67, 81–84].

On the other hand, OA treatment lowers the blood pressure of OSA patients. A network meta-analysis revealed that the average decrease in systolic blood pressure of patients on OA treatment was 2.1 mmHg (95% CI 0.8–3.4 mmHg), while the decrease in diastolic blood pressure was 1.9 mmHg (95% CI 0.5–3.2 mmHg), which are slight but significant decreases in blood pressure [81]. The difference in the antihypertensive effect of OA compared to CPAP, which is known to have an antihypertensive effect on OSA, was − 0.5 mmHg (95% CI − 2.0 to 1.0 mmHg) for systolic blood pressure and − 0.2 mmHg (95% CI − 1.6 to 1.3 mmHg) for diastolic blood pressure, with no significant difference observed. Furthermore, another network meta-analysis confirmed a decrease in blood pressure using a 24-h sphygmomanometer both during the day (systolic blood pressure 2.2 ± 0.7, diastolic blood pressure 1.9 ± 0.6 mmHg) and night (systolic blood pressure 3.8 ± 0.8, diastolic blood pressure 1.8 ± 0.6 mmHg) [85].

Apart from an RCT, an observational study in which cardiovascular death of OSA was set as an endpoint compared 562 severe OSA patients who were prescribed either CPAP or OA, with 208 patients as non-OSA controls, OA reduced cardiovascular death as much as CPAP [hazard ratio 1.08 (95% CI 0.55–1.74, *p* = 0.71)], and the cumulative cardiovascular mortality was significantly higher in the dropout group than in the OA group or CPAP group [86].

In summary, OAs are likely to reduce the onset of cardiovascular disease, along with the resulting mortality rate in the long run. However, at present, in addition to cardiovascular risk-related biomarkers, there are insufficient reports on OSA’s effects on heart rate and heart rate variability, among others, as endpoints; therefore, more methodologically high-quality long-term studies are required.

**c. Side effects of OA **[87]

Although OA is an effective treatment for patients, it has both short-term and long-term side effects. The most common short-term side effects include hypersalivation or decreased saliva production, pain and discomfort in the teeth and gums, occlusal abnormalities when waking up, and discomfort in the jaw muscles as well as temporomandibular joints. These symptoms are generally temporary and often disappear over time. The degree of dental change, including proclination/retroclination of incisors due to long-term use of OA, largely depends on the period of OA use and the amount of mandibular advancement, which often cannot naturally return to the original state.

**CQ 24. Weight loss management for OSA**

**CQ 24–1 Does weight loss management improve apnea in OSA patients? [BQ]**

- Weight loss therapy reduces apnea in OSA patients. [Evidence level: C].

**CQ 24–2 Does weight loss management improve the QOL of OSA patients? [BQ]**

- Weight loss therapy may improve the QOL of OSA patients. [Evidence level: D].

**CQ 24–3 Does weight loss management improve cardiovascular disease risk factors in OSA patients? [BQ]**

- Weight loss may improve risk factors for cardiovascular disease, such as hypertension, diabetes, and dyslipidemia in obese OSA patients. [Evidence level: C].

**Remarks**

Obesity is one of the most important risk factors for OSA and can be improved. Weight loss reduces airway obstruction and apnea by relieving the structural burden on the upper airways.

Many intervention studies, including RCTs, have been conducted on weight loss therapy in OSA patients, with meta-analyses that integrate these results having also been conducted. The results of all meta-analyses indicate that AHI consistently improves with weight loss [88–91].

There are two pillars of weight loss therapy: diet therapy and exercise therapy.

The diet followed in many intervention studies is a structured diet that replaces the diet with liquid supplements early in the intervention [24, 92–96]. While this liquid supplement contains a sufficient amount of essential amino acids, vitamins, and minerals that tend to be deficient during weight loss, it is a prepared diet that minimizes sugars and lipids as energy sources. The dietary composition after liquid supplements varies depending on the study, including those that follow the recommendations of the National Cholesterol Education Program (NCEP) [24] and those that reduce fat to less than 30% of total calories ingested [92, 95]. It is unknown whether a diet with liquid supplements is suitable for OSA patients in Japan because weight loss using liquid supplements is not recognized as a common weight loss method in Japan. In addition, the average baseline body weight of these studied patients exceeds 100 kg, which does not match the patient characteristics of Japan. At this moment, it is recommended to perform weight loss therapy for normal obese patients.

Exercise therapy includes aerobic endurance and muscular resistance exercises. It has been reported that exercise therapy combining aerobic endurance exercise and resistance exercise improves AHI even without weight loss [97]. It is believed that this is because the respiratory muscles were strengthened by resistance exercise, thereby increasing lung volume, indicating that resistance exercises may contribute to the improvement of OSA without weight loss intervention. For this reason, it is recommended that exercise therapy for OSA patients be combined with muscular resistance exercise, in addition to aerobic endurance exercise.

Many studies have indicated that weight loss therapy through lifestyle-related interventions, such as diet and exercise, is effective in improving AHI. However, it is difficult to achieve weight loss of 10% or more via lifestyle intervention alone, so it is not expected that AHI will be sufficiently effective to improve the target value of treatment [98]. Although there are reports that weight loss therapy improved daytime sleepiness, which is a symptom of OSA patients, as well as QOL [95, 97, 99], this has not been sufficiently verified. Therefore, weight loss therapy is not recognized as an independent treatment.

While there is no consistent evidence that weight loss in OSA patients improves risk factors for cardiovascular disease such as hypertension, diabetes, and dyslipidemia [24, 94, 100], most guidelines recommend that obese patients maintain an appropriate weight by correcting lifestyle habits [19, 101, 102]. Even in obese OSA patients, weight loss may improve these risk factors for cardiovascular disease and prevent its occurrence.

Many studies have indicated that weight loss therapy by improving lifestyle-related habits, such as diet and exercise, cannot replace standard treatments such as CPAP, but does contribute to the improvement of AHI. It is important to educate obese OSA patients about lifestyle habits, such as utilizing diet and exercise interventions for weight loss.

**CQ 25. Positional therapy for OSA**

**CQ 25–1 Does positional therapy improve apnea in OSA patients? [BQ]**

- Sleeping in a non-supine position (mainly in the lateral position) may reduce apnea in OSA patients. [Evidence level: D].

**Remarks**

It is known that in approximately half of OSA patients, apnea is exacerbated by obstruction of the pharyngeal airway when sleeping in the supine position, while conversely, apnea is reduced in the lateral position or the half-sitting position with the upper body raised by 30 to 60°. OSA that doubles AHI when the sleeping position changes from the lateral to supine is called positional OSA [103]. Positional OSA patients are often less severe, younger, with lower BMI, and have different characteristics than patients whose AHI does not change depending on their sleeping position.

The purpose of positional therapy is to prevent assuming a supine position during sleep. The effects of positional therapy have been verified by randomized crossover trials or randomized controlled trials, with two meta-analyses existing that integrate these results. These meta-analyses indicate that AHI reduces with positional therapy compared to those without posture therapy, and that positional therapy did not improve apnea as much as CPAP treatment [104, 105].

One classic and well-known positional therapy is the tennis ball technique (TBT). TBT is a method involving making a pocket on the back of a patient’s sleeping garment, in which a tennis ball is placed. This can prevent the patient from assuming a supine position during sleep. There are several studies that have verified the effects of TBT. Jackson et al. reported from a 4-week randomized controlled trial that AHI was significantly reduced in the TBT-treated group compared to the group that received sleep hygiene advice, although daytime sleepiness and blood pressure were not improved [106]. Other randomized crossover studies were not able to observe a significant decrease in apnea during sleep with TBT-treatment compared to CPAP treatment. Although CPAP treatment was more effective than TBT in reducing AHI, there was no significant difference between the two in terms of improving daytime sleepiness and cognitive function [107, 108].

Devices used for positional therapy are being developed overseas. Many devices for positional therapy are in the shape of a chest-worn band, which was developed under the same principle as TBT [108–110]. In addition, new technologically advanced devices have been developed in recent years [111–115]. While these effects have also been verified by randomized crossover studies and randomized controlled trials, most are small-scale. Two large intervention studies on positional therapy are currently underway (US clinical trial registration number [ClinicalTrials.gov]: NCT03061071, NCT02553902).

Positional therapy that maintains a non-supine position during sleep contributes to improved AHI in positional OSA patients, with reduced apnea in non-supine positions. It may be an effective alternative to standard treatment in patients with mild cases or in those with difficulty introducing and maintaining CPAP treatment. However, the devices used for positional therapy have not been standardized. Although it is necessary to verify their effects using a standard device to be established as a treatment method, it is currently used as an axillary treatment because there is no evidence regarding benefits with its use among Japanese patients. It is recommended that mild cases and patients who have difficulty with standard treatment, such as CPAP, be instructed regarding their sleeping position upon confirming that apnea is reduced in the lateral position.

**CQ 26. Oxygen therapy for OSA**

**CQ 26–1 Does oxygen therapy improve the QOL of OSA patients? [BQ]**

- The grounds that oxygen therapy improves the QOL of OSA patients is unclear. [Evidence level: D].

**CQ 26–2 Does oxygen therapy improve hypertension in OSA patients? [BQ]**

- It is not clear whether oxygen therapy is effective in improving hypertension in OSA patients. [Evidence level: C].

**CQ 26–3 Does oxygen therapy improve diabetes in OSA patients? [BQ]**

- The grounds that oxygen therapy improves diabetes in OSA patients is unclear. [Evidence level: D].

**CQ 26–4 Does oxygen therapy improve cardiovascular disease in OSA patients? [BQ]**

- Although there are reports that nocturnal hypoxia predicted cardiovascular-related mortality, there is currently little evidence that it is useful in controlling the onset of cardiovascular disease. [Evidence level: C].

**CQ 26–5 What are the side effects of oxygen therapy? [BQ]**

- Oxygen therapy for OSA prolongs the duration of apneic events, and may lead to hypercapnia. [Evidence level: –].

**Remarks**

**a. QOL improvements**

Oxygen therapy may be a considered a treatment option for OSA patients that are CPAP-intolerant. However, there are few studies on whether oxygen therapy improves the QOL of OSA patients, with no conclusion having been reached as of date. A recent report indicated that 12 weeks of oxygen therapy resulted in an improvement of daytime sleepiness, with a physical component summary (PCS) of health-related QOL (Short-Form 36 scores: SF-36) and PCS improvement reported to be superior to CPAP in OSA patients with coronary artery disease or three or more major risk factors for coronary artery disease [116]. However, contradictory results have been reported with regards to the improvement effects of oxygen therapy on daytime sleepiness and neuropsychological symptoms. Nevertheless, further research results are anticipated [116–121].

**b. Hypertension improvement**

Unlike CPAP, which applies positive pressure to the upper airways, oxygen therapy does not generally have the ability to relieve upper airway obstruction. In other words, oxygen therapy cannot improve AHI [117–119, 122, 123]. The same was observed in children [124]. However, oxygen therapy is known to improve mean nocturnal arterial oxygen saturation (mean SpO_2_) and the degree of decrease in arterial oxygen saturation associated with apnea hypopnea (mean nadir SpO_2_), with the effects thereof being similar to CPAP or greater [117–119, 122, 123].

However, unlike CPAP therapy, oxygen therapy is often reported as having no blood pressure lowering effect, and it has been indicated that pathological conditions other than nocturnal hypoxemia, such as transient arousal reaction (arousal), hypercarbonemia, and respiratory effort-related intrathoracic pressure changes, are involved in the onset and exacerbation of hypertension in OSA patients [119, 122, 125]. On the other hand, a recent double-blind randomized crossover comparative study reported that switching to supplemental oxygen after CPAP discontinuation made it possible to prevent blood pressure from rising again compared to supplemental air [126].

**c. Diabetes improvement**

The effect of oxygen therapy on OSA on glucose metabolism has not been studied, with its effects thereof being currently unclear.

**d. Improvement of cardiovascular disease**

Nocturnal hypoxemia, including intermittent hypoxia associated with apnea hypopnea, plays an important role in the progression of cardiovascular disease in patients with OSA. Therefore, oxygen therapy that improves nocturnal hypoxemia may be useful in suppressing the progression of cardiovascular disease, even if it cannot improve AHI. However, at this time, it has not been clarified whether long-term oxygen therapy has the effect of preventing cardiovascular disease.

On the other hand, it has been noted in recent years that OSA patients have a high risk of perioperative complications. In particular, hypoxemia associated with apnea or hypopnea events are known to be risk factors for postoperative complications, such as arrhythmia and delirium. Thus, it is desirable that oxygen therapy be given to CPAP-intolerant patients [119]. However, especially on the first night following surgery, CO_2_ may increase due to oxygen administration; therefore, it is better to consider overnight percutaneous CO_2_ monitoring [127].

**e. Side effects**

It should also be noted that oxygen therapy prolongs the duration of apnea and hypopnea [119, 128]. This is because long-lasting apneic events not only cause an excessive increase in PaCO_2_ and an abrupt increase in sympathetic nerve activity when breathing is resumed, but also prolong intrathoracic negative pressure due to inspiratory efforts for upper airway obstruction, in addition to potentially increasing adverse effects on the living body, such as an increase in left ventricular afterload due to an increase in transmural pressure and vascular endothelial dysfunction from shear stress.

**CQ 27 Otorhinolaryngological surgery for OSA**

**CQ 27–1 Does otorhinolaryngological surgery improve the QOL of OSA patients? [BQ]**

- Otorhinolaryngological surgery is expected to improve the QOL of OSA patients in certain aspects as a short-term result. In particular, nasal surgery improves QOL, even if AHI does not improve. [Evidence level: C].

**CQ 27–2 Does otorhinolaryngological surgery improve cardiovascular risk factors in OSA patients? [BQ]**

- Although some papers indicate an improvement in the risk of developing cardiovascular disease following surgery, there are no convincing reports. [Evidence level: D].

**CQ 27–3 What are the side effects of otorhinolaryngological surgery? [BQ]**

- With regards to perioperative management after uvulopalatopharyngoplasty (UPPP), it is necessary to pay attention to bleeding and respiratory trouble immediately after the surgery. A few reports indicate that its long-term postoperative side effects include insufficient closure of soft palate after UPPP, pharyngeal discomfort, effects on swallowing, and dysgeusia. Furthermore, no therapeutic effects have been confirmed for uvula-assisted uvulopalatoplasty (LAUP). There are reports of airway stenosis occurring due to wound contractures after the operation. [Evidence level: –].

**Remarks**

**a. Reports on the effect of each surgery**

There was only one positive cohort of single-center RCT designs for UPPP, which reported a decrease in AHI from 53.3 to 21.1 after the 6-month evaluation (52.6 to 46.8 in control) while having no side effects. In the UPPP meta-analysis including this, it was reported that the severity of AHI significantly improved from 35.66 to 13.91, with subjective sleepiness improving from 11.65 to 5.08 after 8 months in selected patients [129–133].

A meta-analysis of trans-oral robotic surgery (TORS) reported that AHI improved from 44.3 to 17.8 (p < 0.01), while ESS improved from 12.9 to 5.8 (p < 0.01) [134].

Although AHI was not significantly different after 4 months, in a small number of single-center RCT-design prospective cohorts of nasal surgery (n = 49: 27 vs. 22), ESS was significantly improved in the surgery group alone. While there is little or no improvement in AHI in the meta-analysis, significant improvement in ESS and QOL were observed [135, 136].

With regards to its effect on CPAP, it was reported that CPAP pressure decreased from 11.6 cm to 9.5 cm, with 89.1% of patients acknowledging improved use of CPAP following surgery, while the usage time increased from 3.0 h to 5.5 h [135].

A meta-analysis of multilevel surgery including UPPP, tongue surgery, and nasal surgery reported a 60.3% decrease in AHI and a 40.3% decrease in ESS, indicating that the effects of CPAP increased CPAP usage rate and use time [129, 131, 137].

There are several reports of single-center RCT prospective cohorts for mild OSA in radiofrequency treatment, with improvements observed in AHI and ESS [131, 138].

A multicenter RCT prospective cohort for hypoglossal nerve stimulation reported significant improvement in AHI, oxygen desaturation index, ESS, and FOSQ at the 12th and 18th months, with improvement in subjective symptoms, such as ESS and FOSQ, reported even at the 24-month mark [139].

A meta-analysis of LAUP indicated that the surgical effect group accounted for only 23% of AHI improvements, with 44% of patients reporting worsened AHI. In addition, side effects were observed. Thus, it is not recommended as a treatment for OSA [131, 138, 140].

Tracheostomy performed in selective cases improved AHI from 92.0 to 17.3. However, the decrease in QOL with the surgery needs to be considered [129, 130, 141].

**b. Effects of otorhinolaryngological surgery on systemic diseases**

There are few reports of the effects of otorhinolaryngological surgery on systemic diseases. There are only limited short-term reports of improvement in hypertension in OSA patients with UPPP.

**c. Reports on side effects**

There are almost no reports of the side effects of otorhinolaryngological surgery [129, 132]. A meta-analysis indicated a complication rate of 1.5%, a mortality rate of 0.2%, and a rate of anesthesia-related events of 12.5%. With regard to LAUP, a surgical effect group only accounted for 23%, with 44% of patients experiencing worsened AHI and 1 to 12% suffering narrowing of the airway due to wound contracture as a complication. Therefore, it is not recommended as a treatment for OSA [129, 131, 140].

**CQ 28. Maxillofacial plastic reconstruction treatment for OSA**

**CQ 28–1 Does maxillofacial plastic reconstruction surgery improve the QOL of OSA patients? [BQ]**

- For cases in which CPAP and OA cannot be used, improvements in AHI, snoring following surgery, and QOL can be expected due to changes in facial morphology. [Evidence level: D].

**CQ 28–2 Does maxillofacial plastic reconstruction surgery improve cardiovascular disease risk factors in OSA patients? [BQ]**

- Although some papers indicate an improvement in the risk of developing cardiovascular disease following surgery, there are no highly accurate reports on the subject. Thus, is not clear whether or not it improves the risk of developing cardiovascular disease. [Evidence level: D].

**CQ 28–3 What are the side effects of maxillofacial plastic reconstruction surgery? [BQ]**

- Frequent cases include lower lip dysesthesia, with postoperative neuropathic pain also observed in some cases. In rare cases, occlusal abnormalities, postoperative serious heart disease and airway stenosis, among others, are observed. [Evidence level: –].

**Remarks**

**a. What is maxillofacial plastic reconstruction surgery?**

Maxillo-mandibular advancement (MMA) and genioglossus and geniohyoid muscles advancement (GA) have been used as a treatment for OSA since around 1980. This MMA is performed by Le Fort type 1 osteotomy for the maxilla (horizontal osteotomy of the maxilla), with sagittal splitting ramus osteotomy (SSRO) performed for the mandible. It is a technique involving separation of the upper and lower bones (the part where the teeth are implanted) and moving them forward. As a result, in addition to expanding the oral volume, the soft tissue attached to the mandible is pulled and the upper airway is dilated [142]. Furthermore, GA pulls the tongue upward, resulting in dilation of the upper airway. It is expected that the mucosal part of the pharyngeal airway becomes tense, and with the added traction of soft tissue, the collapse of the upper airway is expected to improve.

The Sleep Disorders Center at Stanford University in the United States, a pioneer institution in sleep surgery for OSA including MMA, has proposed a two-step treatment policy [143], which is followed by many facilities. First, Phase 1 includes intranasal surgery, soft palate pharyngoplasty, genioplasty, and radiofrequency surgery on the base of the tongue (tongue contraction), which are relatively minimally invasive surgeries. Should the effect thereof be insufficient, hard tissue surgery such as MMA/Phase 2 is considered. Health insurance covers this surgery as a surgical orthodontic treatment in Japan.

**b. Improvement of cardiovascular disorders after MMA**

Although some papers indicate an improvement in the risk of developing cardiovascular disease following surgery, the number thereof is small, making it difficult to come to a definitive conclusion. Furthermore, the accuracy of the reported papers is not high, and it is not clear at present whether or not they will be improved.

**c. Side effects of MMA**

As side effects of MMA:

1. With regard to perioperative complications, there may be an existing risk of heart disease specific to OSA patients, with 1.0% of serious complications including cardiac arrest and arrhythmia.
2. Facial dysesthesia (14.2%) and postoperative neuropathic pain.
3. Occlusal abnormality (up to 44%).

The above have been reported as complications [144]. Although the frequency of surgical orthodontic surgery, which is a similar procedure, is low, complications such as severe postoperative airway obstruction and blindness have also been reported.

**d. Effectiveness of MMA**

An RCT comparing the effectiveness of MMA and auto-titrating CPAP in 50 OSA patients found that there was no difference between the two groups in terms of AHI and ESS approximately 1 year following surgery [144]. This is the only RCT on the subject, with many other studies on the therapeutic efficacy of MMA existing based on observational studies [131, 145, 146]. With regards to the therapeutic efficiency of MMA, a meta-analysis of these observational studies found that AHI decreased from an average of 63.9 to 9.5 with an average follow-up period of 5 months, and that an AHI < 5 was achieved in 43.2% of patients [145]. Although a young age, low pretreatment AHI, low BMI, and degree of anterior movement of the maxilla are cited as predictors of effectiveness of MMA, there are no high-quality studies on these factors to date [145, 147]. We ultimately weakly recommend maxillofacial plastic reconstruction surgery for the treatment of OSA, because of the low level of evidence on the subject.

**CQ 29. CPAP use time**

**CQ 29–1 Does CPAP use time in OSA patients affect its therapeutic effects? [BQ]**

- CPAP use time affects its therapeutic effects on OSA patients. [Evidence level: B].

**CQ 29–2 How many hours of CPAP treatment are required to improve daytime sleepiness in OSA patients? [BQ]**

- CPAP treatment for longer than 4 h per night is required to improve subjective daytime sleepiness. It is desirable to use it daily to maintain its effects. [Evidence level: B].

**CQ 29–3 How many hours of CPAP treatment are needed to improve the incidence of hypertension and cardiovascular events in OSA patients? [BQ]**

- CPAP treatment for at least 4 h per night is required to reduce the frequency of hypertension and cardiovascular events. [Evidence level: A].

**Remarks**

In relation to life prognosis due to CPAP use, it has been reported that treatments of less than 1 h per night are ineffective, with a difference observed compared with groups averaging 4 h or more [148]. A treatment duration of 4 h or more improves subjective daytime sleepiness [149] and reduces the frequency of hypertension and cardiovascular events [16, 150, 151]; 6 h or more to improve wakefulness maintenance time [149]; and 7.5 h to improve health states (SF-36) [149]. Furthermore, it has been reported that OSA reappears immediately after discontinuation of treatment, with subjective sleepiness and increased blood pressure in the morning observed 2 weeks later [48, 152, 153].

It has been reported that even less than 4 h can be effective for OSA with cardiovascular disease, which is considered relatively less likely to cause sleepiness when focusing only on subjective daytime sleepiness [77]. However, the precise duration in terms of how many hours has not been established, as the required sleep time varies from person to person. However, considering its effects on glucose tolerance and prognosis, 4 h or more is considered desirable.

**CQ 30. Improvement of adherence**

**CQ 30–1 Is there any difference in CPAP adherence between fixed pressure CPAP and auto CPAP in the treatment for OSA? [BQ]**

- If the pressure is appropriately set, there is no difference in CPAP adherence between fixed pressure CPAP and auto CPAP. [Evidence level: C].

**CQ 30–2 Does the pressure relief function improve CPAP adherence? [BQ]**

- The effect of improving CPAP adherence using the pressure relief function has not been statistically recognized. [Evidence level: C].

**CQ 30–3 Does choosing the optimal mask for the patient improve CPAP adherence? [BQ]**

- Choosing an optimal mask for each patient may improve CPAP adherence. [Evidence level: C].

**CQ 30–4 Does the use of humidifiers and nasal drops improve CPAP adherence? [BQ]**

- In the case of nasal congestion, the use of humidifiers and nasal drops may improve CPAP adherence. [Evidence level: C].

**CQ 30–5 Do patient education and treatment interventions improve CPAP adherence? [BQ]**

- Supportive interventions, educational interventions, behavioral therapies, etc. may improve CPAP adherence. [Evidence level: C].

**Remarks**

For this particular CQ, “CPAP” pertains to fixed pressure CPAP.

**a. CPAP device settings**

There is no difference between the therapeutic effect of Auto CPAP (APAP) titration and conventional CPAP manual titration. Taking into consideration the cost of manual titration, APAP is recommended as an alternative to manual titration, with no difference in adherence [154]. While APAP was used 12 min longer in the 2009 APAP and CPAP RCT crossover study [155], there was no significant difference observed in terms of adherence. According to a meta-analysis in 2012 [156], APAP usage time was 11 min longer than CPAP, which was statistically significant. However, it employed a short-term follow-up; thus, the difference in terms of its therapeutic effect is unknown.

According to a 2011 systematic review and meta-analysis [157], the difference in the use time of the PAP device between EPR users and non-users was 0.16 h in the parallel test and 0.2 h in the crossover test, which was not a significant difference. On the other hand, a 2015 report [158] indicated that adherence was improved using EPR from the start of CPAP for SAS with high nasal resistance.

**b. Interfaces such as masks**

Masks are roughly divided into nasal masks and full-face masks, with the use of nasal masks recommended during the CPAP introduction period. There are many studies comparing the two, and it has been reported that the nose mouth mask increases residual AHI, leakage, and CPAP required pressure [159]. However, there are very few reports comparing adherence between the two in many cases.

Although similar nasal masks have been devised, such as the pillow type, upon examination of a normal nasal mask and a pillow mask, it was reported that there was no significant difference in the usage time of the pillow mask despite its usage rate being higher [160].

It has been reported that the use of a humidifier improved nasal congestion and inflammation of the nasal mucosa in patients with nasal congestion [161]. A double-blind study looking at steroid nasal drops and a placebo was performed on OSAS patients with or without symptoms at the same time as CPAP treatment, which found that there was no significant difference in terms of improvement of rhinitis symptoms or CPAP adherence [162]. Furthermore, a comparison among the three groups of dry CPAP, humidifier CPAP, and dry CPAP + nasal drops found that the only significant difference was the improvement of rhinitis symptoms by the humidifier, with no difference in adherence among the three groups [163].

Previous APSS practice parameters (2006) and The Cochrane Library (2013) [165] recommend the use of topical steroids [164]. However, based on Reference number 10, many recent papers do not recommend the use of steroid nasal drops.

**c. Treatment interventions such as patient education**

The guidelines published in 2009 by AASM for long-term treatment management of OSA mention the importance of having patients understand the functions of PAP devices, its precautions for use, maintenance methods, and usefulness of PAP therapy, among others, including teaming up for patients to provide an optimal interface. In particular, intervention in the first few weeks following the introduction of treatment is most important.

According to the 2014 Cochrane Database Systematic Review [166], it has been reported that CPAP usage increased with supportive interventions, such as encouraging patients, and that CPAP usage rates were significantly improved by short-term educational interventions and behavioral therapy.

In recent years, there have been reports verifying the effects of telemedicine [167]. It is believed that the combined use of telemonitoring and telemedicine education have improved adherence the most.

**CQ 31. Recurrence of OSA due to withdrawal of CPAP treatment**

**CQ 31–1 Will OSA recur after withdrawal of CPAP treatment? [BQ]**

- CPAP withdrawal does not worsen AHI compared to before treatment, but OSA does recur. [Evidence level: A].

**Remarks**

A study in which CPAP was discontinued for 2 days among patients who had been using CPAP for 4 months or more found that apnea was low and hypopnea/RERA high, and in severe cases, AHI and decreased oxygen saturation were improved compared to before CPAP use. There were no changes in their scores on the Stanford sleepiness scale, MSLT, and psychomotor vigilance test [168].

A report on CPAP users in which CPAP was discontinued for 2 weeks indicated that OSA recurred within a few days, with symptoms such as subjective sleepiness again observed. However, their psychomotor function did not deteriorate. Although they found a decline in endothelial function, with increases in early-morning blood pressure, heart rate, and urinary catecholamines, no changes were found in terms of systemic inflammatory markers, insulin resistance, or lipids [48].

A 4-day withdrawal of CPAP demonstrated that 71% of cases had 4% ODI of 10 or higher upon measurement with an oximeter, with 45% of patients in 10 or less remaining after 14 days of CPAP withdrawal. ODI after 4 days was associated with originally high ODI, long-term CPAP use, smoking, and a large neck circumference [169]. Although overnight CPAP withdrawal reduces driving ability, subjective sleepiness is associated with the EEG evaluation of sleepiness, with patients being aware of sleepiness due to CPAP withdrawal [170]. According to a report examining the results of driving simulation, improvement was seen within a few days after the introduction of CPAP, with this improvement maintained 7 days after withdrawal; however, the difference from the control group was reduced [153]. Studies on modafinil administration at the time of CPAP withdrawal indicated improved its positive effects in terms of sleepiness during a driving simulation [171].

A meta-analysis examining the effects of CPAP withdrawal on blood pressure from four reports found that systolic blood pressure taken in the morning at the clinic increased by 1.1 mmHg; however, diastolic blood pressure did not change, and there was no significant difference observed versus blood pressure taken at home [172]. With regard to CPAP usage status, during the Great East Japan Earthquake, a survey within 14 days indicated that 92.3% of 1,047 cases were unable to use CPAP due to power outages due to concerns about aftershocks, relief activities, and loss of equipment, among others. Daytime sleepiness, insomnia, and headache recurred in 25% of 966 cases, indicating the need for measures for continuous use of CPAP in the event of a major disaster [173].

**CQ 32. Use of hypnotics**

**CQ 32–1 Do you prescribe sleeping pills for insomnia in OSA patients? [BQ]**

- Sleeping pills should not initially be used in the treatment of OSA patients with insomnia. The treatment of OSA itself should be prioritized. [Evidence level: C].

**CQ 32–2 What are the side effects of hypnotic treatment for insomnia in OSA patients? [BQ]**

- The results differ depending on the medicine; however, an increase in the number of events, along with extension of the event time, have been reported, particularly in severe cases. [Evidence level: –].

**CQ 32–3 Are sleeping pills effective for improving adherence in patients using CPAP? [FQ]**

- If insomnia is present despite the proper use of CPAP, we suggest the use of sleeping pills. [Strength of recommendation: 2 (Consensus rate: 100%)] [Evidence level: C].

**CQ 32–4 What are the side effects of hypnotic treatment for CSA? [BQ]**

- While reports on the number of events before and after use for CSA, sleep construction and sleepiness, among others, do exist, although this require further investigation due to the minimal amount of studies on the subject. [Evidence level: –].

**Remarks**

**a. Insomnia treatment and sleeping pills for OSA**

Insomnia is an insurance indication for the prescription of sleeping pills, but if used for the treatment of OSA itself, it will not be covered by insurance.

It is said that the prevalence of insomnia in OSA patients ranges from 39 to 55%, and that sleeping pills are often taken because insomnia adversely affects CPAP adherence. Problems with the use of sleeping pills may include the aggravation of OSA, such as a decrease in respiratory events due to decreased upper airway hypotonia and ventilatory response, along with prolonged respiratory event time [174, 175].

A randomized controlled trial of triazolam and a placebo conducted in severe OSA patients found a significant increase in hypopnea/apnea time and a decrease in minimum SpO_2_ during non-REM sleep [176].

It has been found that ramelteon has no muscle relaxant effect, and can be taken relatively safely.

Randomized controlled trials using flurazepam, temazepam, nitrazepam and zolpidem, and ramelteon have led to improvements in sleep, with no significantly adverse effects on OSA [177, 178].

A randomized controlled trial using placebo and nitrazepam in mild to moderate OSA patients, found that the nitrazepam group exhibited an increase in total sleep time, with no worsening AHI and minimum SpO_2_ [179].

There is still little research on suvorexant, which is an orexin receptor antagonist. However, a study of 26 patients with mild to moderate OSA patients who received 40 mg of the drug, which is twice the usual dose in the United States and Japan, reported that the decrease in AHI and mean SpO_2_ was clinically very mild [180].

However, while many studies involve mild to moderate OSA patients, it is important for severe OSA patients to take sleeping pills for control, along with treatment such as CPAP (refer to next section “b. CPAP Treatment and hypnotics”).

**b. CPAP treatment and hypnotics**

Randomized controlled trials of a placebo and zolpidem in severe OSA patients receiving CPAP therapy have indicated that zolpidem does not diminish the therapeutic effect of CPAP [181], with reports also indicating that the use of sleeping pills improved sleep parameters and CPAP adherence during titration.

A study in which eszopiclone was administered to OSA patients to compare its effect with a placebo found that the eszopiclone group exhibited an increase in total sleep time and sleep efficiency, a decrease in sleep latency and interrupted sleep time, and residual AHI [182], while another randomized controlled trial of a placebo and eszopiclone in OSA patients found that the group receiving eszopiclone during CPAP titration had significantly higher CPAP usage and overnight usage time 4–6 weeks after CPAP introduction [183].

A randomized controlled trial of eszopiclone and placebo in severe OSA patients indicated that the eszopiclone group had significantly higher CPAP usage [184].

As mentioned above, the combined use of CPAP and sleeping pills is considered to be effective for improving compliance.

**c. CSA/CSB treatment and sleeping pills**

The indication for sleeping pills is insomnia; thus, if sleeping pills are used as a treatment for CSA and CSB, they will not be covered by insurance.

There are several reports on patients with central sleep apnea. Sleeping pills reduced the number of respiratory events as well as improved sleep architecture and subjective symptoms, such as sleepiness [185–188]. While sleeping pills have the potential to reduce respiratory events for CSA and CSB, the number of reports on the subject is small; thus, further studies are needed.

**CQ 33. CSB treatment and indications**

**CQ 33–1 What treatments are available for CSB? [BQ]**

1. Treatments for underlying diseases associated with the development of CSB, such as drug therapy for heart failure and pacemakers including cardiac resynchronization therapy (CRT), are treatments for CSB itself. [Evidence level: B].
2. Treatments that directly suppress CSB, including CPAP, bi-level PAP, ASV, and oxygen therapy. [Evidence level: B].

**CQ 33–2 What type of CSB patients should be treated with CPAP? [FQ]**

- For CSB associated with cardiovascular disease such as heart failure, CPAP is recommended if the CSB remains at a moderate or higher level even after treatment of the underlying disease is optimized. [Strength of recommendation: 2 (Consensus rate: 100%)] [Evidence level: B].

**CQ 33–3 What CSB patients should be treated with ASV? [FQ]**

1. ASV can be considered if CPAP is intolerable or if AHI ≥ 15 on CPAP for patients with moderate-to-severe CSB having left ventricular ejection fraction (LVEF) > 45% remaining after optimization of heart failure treatment in those with NYHA functional classification III or higher. [Not recommended] [Evidence level: B].
2. ASV treatment can be considered, if further treatment is needed, in the event that CPAP is intolerable or AHI ≥ 15 on CPAP for patients with moderate-to-severe CSB having predominant central respiratory events remaining after the optimization of heart failure treatment in symptomatic heart failure of LVEF ≤ 45% (NYHA cardiac function classification III or higher). [Not recommended] [Evidence level: C].

**CQ 33–4 Which subset of CSB patients should be treated with oxygen therapy? [FQ]**

- Oxygen therapy is suggested, if CPAP or ASV is intolerable, for patients with moderate-to-severe CSB remaining after optimization of heart failure treatment in symptomatic heart failure of LVEF ≤ 45% (NYHA cardiac functional classification III or higher). [Strength of recommendation: 2 (Consensus rate: 100%)] [Evidence level: B].

**CQ 33–5 Which subset of CSB patients should be treated with drug therapy? [FQ]**

- It is recommended that CSB patients with heart failure undergo drug treatment or have their current drug regimens optimized in accordance with the Japanese heart failure clinical practice guidelines. [Strength of recommendation:1 (Consensus rate: 100%)] [Evidence level: B].

**CQ 33–6 Which subset of CSB patients should be treated with a device (pacemaker)? [FQ]**

- A pacemaker is recommended, based on the indication for the treatment of heart failure, if CSB patients with heart failure are indicated for its use. In particular, CRT can be expected to improve CSB. [Strength of recommendation:1 (Consensus rate: 100%)] [Evidence level: B].

**Remarks**

Since most CSBs are caused by pulmonary congestion (hyperventilation and hypocapnia), enhanced ventilatory response, and prolonged circulation time due to low cardiac output, it is important that CSB patients be screened for heart failure, and if so, receive optimize treatment for it [189, 190]. If CSB remains even after that, oxygen therapy, CPAP, bi-level positive airway pressure (bi-level PAP), and ASV can be considered as direct CSB treatments [189–193]. Although drugs such as acetazolamide and theophylline, along with carbon dioxide inhalation, have also been reported to be effective in suppressing CSB, it is not recommended due to lack of studies assessing its safety for long-term use [189–193].

The inhibitory effect of CSB increases in the order of oxygen therapy, CPAP, bi-level PAP, and ASV [194]. The CANPAP trial, which is a randomized controlled trial in which patients with AHI ≥ 15 and LVEF ≤ 45% who have predominant central respiratory events were enrolled, found that there was no difference in prognosis between patient with and without CPAP; however, approximately half of the CPAP group continued to have AHI ≥ 15 [189–193]. In the post hoc analysis of this CANPAP study, it was reported that the prognosis of patients with CPAP who had AHI < 15 was significantly better than that of the control group, while the prognosis of patients who continued to have AHI ≥ 15 was somewhat worse than that of the control group [195]. Therefore, because sufficient suppression of CSB may lead to improved prognosis, the effectiveness of bi-level PAP and ASV, which can suppress CSB more effectively, has been investigated [189–195]. Although improvement of LVEF in a short term has been reported, bi-level PAP [196] is rarely used in clinical practice due to difficulty in its setting and the lack of data on long-term prognosis. ASV is superior to CPAP and the untreated control group in terms of short-term improvement of cardiovascular parameters and prognosis of approximately 6 months to 1 year [189–192]. The SERVE-HF trial, which is a multicenter, randomized controlled trial, included patients with LVEF ≤ 45% and AHI ≥ 15 experiencing predominant central respiratory events. Although there was no significant difference between the two groups in terms of the primary endpoint, ASV significantly increased all-cause mortality and cardiovascular mortality, which were secondary endpoints [197, 198]. Based on these results, the guidelines of the United States and European Cardiology Societies do not recommend ASV for patients with chronic heart failure (LVEF ≤ 45%) with AHI ≥ 15 having predominantly central respiratory events. Taking into consideration that statements have been issued by the Japanese Circulation Society in conjunction with the Japanese Heart Failure Society, including the latest second report which does not demonstrate research results that raise concerns about safety in Japan, where ASV was used more frequently than in other countries, along with the fact that the medical care system for ASV patients is different from other countries, the ASV treatment for patients who meet the subjects of the SERVE-HF study is not contraindicated, but is considered to be discreet [199]. It is also stated that the necessity of ASV among patients who meet the subjects of the SERVE-HF study should be examined each time, without continuing ASV indiscriminately after observing an improvement in or stabilization of heart failure.

Taking into consideration that cases with AHI < 15 in the CPAP group had a better prognosis than the control group in a CANPAP study [195] and CPAP is cheaper than ASV, and so on, CPAP should be introduced to patients with chronic heart failure who have moderate-to-severe CSB. A change to ASV may be considered for those patients suffering from chronic heart failure with LVEF ≤ 45% (NYHA functional classification III or higher) and CSB patients with moderate or higher central respiratory events, as well as patients receiving CPAP treatment but experiencing tolerance. Patients with LVEF > 45% with CSB and patients suffering from chronic heart failure with CSB, with obstructive respiratory events, were not included in the SERVE-HF study. Therefore, ASV can be considered for cases with NYHA functional classification III or higher, as well as moderate-to-severe CSB. Oxygen therapy may be considered in patients suffering from chronic heart failure with NYHA functional classification III or higher, who have moderate-to-severe CSB that persists after optimization of heart failure treatment, and are intolerant of CPAP or ASV.

Since drug therapy for chronic heart failure, especially ACE inhibitors and β-blockers, can also treat CSB itself, drug treatment for heart failure and optimization thereof should be performed in accordance with heart failure guidelines [189–193]. Furthermore, it has been reported that pacemaker treatment, particularly CRT, can be a treatment for CSB itself; however, if there is an indication as a treatment for heart failure, it will be examined accordingly [189–193].

**CQ 34. CPAP** **,** **ASV, and oxygen therapy for CSB**

**CQ 34–1 Does CPAP improve the QOL of CSB patients? [BQ]**

- CPAP does not improve the QOL of CSB patients. [Evidence level: C].

**CQ 34–2 Does ASV improve the QOL of CSB patients? [BQ]**

- ASV improves the QOL of CSB patients, although minimal. [Evidence level: C].

**CQ 34–3 Does CPAP improve cardiovascular parameters and prognosis in CSB patients? [BQ]**

1. Several months of CPAP improves cardiovascular parameters, such as LVEF and impaired exercise tolerance, in CSB patients with heart failure. [Evidence level: B].
2. Prognosis may be improved in patients whose CSB is suppressed to less than moderate by CPAP. [Evidence level: C].

**CQ 34–4 Does ASV improve cardiovascular parameters and prognosis in CSB patients? [BQ]**

1. Several months of ASV improve cardiovascular parameters such as LVEF and impaired exercise tolerance in CSB patients with heart failure. [Evidence level: B].
2. ASV may improve the prognosis of CSB patients. However, it should be noted that LVEF ≤ 45% may lead to a worse prognosis. [Evidence level: C].

**CQ 34-5 What are the side effects of CPAP for CSB patients? [BQ]**

- Interface discomfort, dryness, as well as skin and eye discomfort may occur. [Evidence level: –].

**CQ 34–6 What are the side effects of ASV for CSB patients? [BQ]**

- Interface discomfort, dryness, as well as skin and eye discomfort may occur. It should be noted that LVEF ≤ 45% may lead to a worse prognosis. [Evidence level: –].

**CQ 34–7 Does oxygen therapy improve cardiovascular parameters and prognosis in CSB patients? [BQ]**

1. Oxygen therapy may improve cardiovascular parameters, such as impaired exercise tolerance, in CSB patients with heart failure. [Evidence level: C].
2. Oxygen therapy has not demonstrated improvement in the prognosis of CSB patients. [Evidence level: B].

**CQ 34–8 What are the side effects of oxygen therapy for CSB patients? [BQ]**

- It may prolong the duration of respiratory events because it weakens the respiratory resumption stimulus due to hypoxia release and improvement. [Evidence level: –].

**Remarks**

Although there are little data on the improvement of QOL by CPAP for CSB, there was no improvement in QOL by CPAP in the CANPAP study [200]. A small randomized controlled trial comparing ASV with CPAP in heart failure patients with CSB indicated poor improvement in QOL with CPAP compared to ASV [201–203]. Some small randomized controlled trials comparing ASV with untreated controls or CPAP have indicated improved QOL with ASV [201–204]. However, despite a wide variety of indicators in the meta-analysis, the results did not improve QOL at any rate [203]. Furthermore, in the SERVE-HF study, while multiple QOL indicators were used as secondary endpoints, none of them improved ASV compared to the control group [204].

Several randomized controlled trials of patients suffering from chronic heart failure with LVEF ≤ 45% indicated improved LVEF, decreased sympathetic nerve activity, and improved exercise tolerance as effects of CPAP on CSB [195, 197, 205, 206]. Similarly, ASV has been shown to improve LVEF, decrease sympathetic nerve activity, and improve exercise tolerance as effects on cardiovascular parameters in a randomized controlled trial of patients suffering from chronic heart failure with LVEF ≤ 45% [195, 197, 205, 206], and even in patients suffering from chronic heart failure with LVEF > 45%, ASV has been shown to improve both the left ventricular diastolic function and exercise tolerance [197].

Regarding the prognosis improvement effect of CPAP, a limited but constant effect has been demonstrated. In a randomized controlled trial of patients suffering from chronic heart failure with LVEF ≤ 45% in a small single center, it was indicated that the prognosis was good in the subgroup with CPAP adherence, while in a post hoc analysis of the CANPAP trial, which is a randomized controlled trial in which patients suffering from chronic heart failure with AHI ≥ 15 and LVEF ≤ 45% were assigned to a CPAP group and a control group, prognosis was evaluated with predominance of central respiratory events, where it was found to be better in the CPAP case with AHI < 15 than that of the control group [195, 197, 203–206]. With regards to the prognosis improvement effect of ASV, in addition to the results of multiple observational studies in which the prognosis of patients suffering from chronic heart failure with LVEF ≤ 45% who were introduced with ASV was relatively good [195, 197, 203–206], small single-center randomized controlled trials of patients with LVEF > 45% of chronic heart failure indicated improved prognosis [197]. However, the SERVE-HF trial, a multicenter randomized controlled trial of patients suffering from chronic heart failure with LVEF ≤ 45%, indicated that the primary endpoint was not significantly different between the two groups, with the secondary endpoints indicating a significant increase in all-cause mortality and cardiovascular mortality in ASV [197, 204]. Based on these results, the guidelines of the Cardiovascular Societies in the United States and Europe do not recommend ASV for patients suffering from chronic heart failure (LVEF ≤ 45%), with AHI ≥ 15 predominantly in central respiratory events. Since ASV was used more frequently than in other countries, along with the fact that the medical care system for ASV patients is different from that of other countries, the ASV for patients who meet the subjects of the SERVE-HF study is not contraindicated, but should be considered cautiously. At present, ASV can be introduced for the purpose of improving the QOL and cardiovascular parameters of patients suffering from chronic heart failure with LVEF ≤ 45%, who have moderate-to-severe higher CSB with predominant central respiratory events; however, introducing ASV as a treatment for CSB in the hope of improving their long-term prognosis needs to be carefully considered. Although the side effects of CPAP and ASV on CSB are minor, such as discomfort due to the interface, dry nasopharynx, and discomfort in the skin and eyes [67], careful considerations should be given to increasing mortality in the SERVE-HF trial when treating ASV in patients suffering from chronic heart failure with LVEF ≤ 45% [197, 204].

Although oxygen therapy is inferior to CPAP and ASV, it has a suppressive effect on CSB, and has been indicated as having the potential to improve cardiovascular disorders such as exercise tolerance; however, its prognosis improvement effect has not been clearly demonstrated, and is limited to the improvement effect of heart failure symptoms [197, 204–206]. On the other hand, there is almost no discomfort in the interface or nasopharynx, and it is easy to use. However, care should be taken as it may prolong the duration of respiratory events, because of the weakened respiratory resumption stimulus due to hypoxia release and improvement.

**CQ 35. Operating a vehicle and the risks thereof**

**CQ 35–1 What type of OSA drivers need to pay special attention to the risks associated with driving? [BQ]**

- The risk of driving accidents increases due to OSA morbidity. In particular, OSA drivers who have moderate-to-severe sleepiness or who have recently experienced irresistible sleepiness, fatigue, as well as inadvertent vehicular accidents or near misses should receive treatment. [Evidence level: C]

**CQ 35–2 Is OSA treatment related to reducing the risk of accidents? [BQ]**

- CPAP treatment reduces the risk of accidents in OSA patients. [Evidence level: B].

**Remarks**

There has been no dispute that dozing off while driving is an important cause of vehicular accidents, with studies reporting that dozing while driving was involved in 15–33% of fatal collisions [207]. Since OSA can cause daytime sleepiness, the risk of vehicular accidents in drivers affected with OSA has been noted since 1980s [208–214], with guidelines or manuals having been prepared in each country to reduce the driving risk of OSA patients [215–218].

Some meta-analyses have assessed the risk of driving accidents in OSA patients [208–211]. Among these, Ellen et al. found no clear accident risk associated with OSA in professional drivers; however, a significant increase in accident risk was confirmed in non-professional drivers. Another meta-analysis revealed that aside from non-professional drivers, professional drivers are also at an increased risk of driving accidents due to OSA morbidity [212]. Overall, the surveys regarding this topic have weaknesses (many relying on self-reports to determine whether or not they have had an experience of causing accidents and inclusion of many non-professional drivers, among others); however, there seemed to be minimal differences in their results. Therefore, the risk of driving accidents is thought to increase in OSA patients [213]. However, although the subjective sleepiness level evaluated by the severity of respiratory disorder (AHI) and the score of ESS manifesting the severity of subjective sleepiness are considered to be associated with accident risk in some studies, the relevance has not reached a significant level. While it has also been reported that the degree of obesity, which is strongly associated with OSA pathology, can be a predictor of accident risk [210], obesity is a phenomenon that is also seen in the non-OSA population, although its specificity in estimating accident risk is thought to be low. Accordingly, development of a screening method which enables for more accurate detection of OSA would be desirable for reducing the risk of accidents [214]. Since there is no definitive indicator for predicting accidents in OSA drivers, in the experience-based guidelines of the American Thoracic Society defined high-risk drivers as those who have moderate-to-severe sleepiness (the level at which unintentional inadequate doze occurs during daily activities) or who have recently experienced irresistible sleepiness, fatigue, as well as inadvertent accidents or near misses [215], with such drivers needing to be warned about driving risks if untreated. This guideline also recommended that high-risk drivers be diagnosed using polysomnography (PSG) as early as possible (targeted within one month), which is almost the same indication written in the Canadian guidelines [216]. In addition, EU guidelines recommend that moderate-to-severe OSA patients should not drive until proper medical management has been given [217]. As described above, because objective indicators of accident risk for OSA drivers have not been sufficiently established, it is necessary for the physician in charge to carefully examine the symptoms and problems related to driving.

With regard to the changes in driving risk after OSA treatment, there are multiple meta-analytic studies for CPAP alone [211, 212]. The actual frequency of accidents, frequency of near misses, and the number of collisions on the simulator were reduced by CPAP treatment. In particular, it has been reported that the effect of suppressing near misses after treatment was clear in cases with a high frequency of accidents before treatment [212]. However, it should be noted that there are drawbacks in these treatment studies, the observation period was not controlled, and the driving frequency/distance of OSA-affected drivers, along with the difference in the effect between professional and non-professional drivers, were not known. Even if the risk of accidents with OSA drivers is reduced by CPAP treatment, the following points should be clarified: (1) how frequent this treatment should be used; and (2) how long it should take to determine the effect of treatment [219]. Although sleepiness remains in 2–6% of cases after CPAP treatment, among OSA cases [220], there are no studies assessing the post-treatment driving risk.

With regards to treatments other than CPAP (oral appliance, surgical treatment, etc.), there have been no reports systematically examining changes in accident risk after treatment.

OSA drivers are likely to have a higher risk of driving accidents than the general population. For patients with moderate-to-severe sleepiness or who have recently experienced an accident or near miss due to sleepiness, diagnostic examination of the disorder should be proactively conducted. CPAP should be performed for patients who are considered to have a high risk of driving accidents after OSA diagnosis. In addition, its effect on the suppression of respiratory events as well as treatment adherence should be objectively confirmed, and their accident risk status should be confirmed by interviews done on a regular basis. It is also desirable to check whether there are any problems with sleep hygiene, and appropriate guidance should be provided if necessary.

**CQ 36. Remote monitoring for CPAP**

**CQ 36–1 Does remote monitoring guidance improve CPAP adherence? [BQ]**

1. Improvement of CPAP adherence can be expected by remote monitoring guidance. [Evidence level: C]
2. Remote monitoring guidance can be expected to reduce the burden on the medical staff and improve convenience on the patient side. [Evidence level: C]

**Remarks**

**a. CPAP treatment and remote monitoring**

The most popular telemedicine in the current field of sleep medicine is remote monitoring of CPAP treatment. With CPAP remote monitoring, daily CPAP usage and treatment data are automatically transferred from a home communication terminal to a data server on the cloud. By accessing the data server, healthcare professionals can view these data at any time as needed. On the other hand, patients can also access the server from mobile apps to check their treatment status.

**b. Expected effects of CPAP remote monitoring**

The following are expected effects of remote monitoring: (1) improved medical access and reduction of hospital visits; (2) improved CPAP treatment adherence (medical staff: early intervention is possible for patients with poor adherence; patients: self-understanding of treatment status and improvement of treatment motivation and self-efficacy, with a self-solving support function when problems occur); (3) improved cost-effectiveness (medical staff: streamlining data management, reducing data collection labor, and shortening consultation time; patients: reducing hospital expenses, consultation costs, and consultation time).

Randomized controlled trials found that using remote monitoring in CPAP introduction period not only improves medical access, but also achieves mostly either: (1) improved CPAP adherence through early intervention or self-management or (2) reduced medical costs and consultation-related effort while maintaining adherence [221–225].

CPAP medical care in Japan is based on frequent face-to-face medical care, which is different from the CPAP insurance system in overseas countries. The usefulness of remote monitoring in Japanese OSA patients with long-term CPAP use was examined in a recent randomized controlled trial comparing three intervention groups, with follow-up done every 3 months and a monthly telemedicine intervention, as well as a face-to-face follow-up every month or every 3 month. This study revealed that 3-month remote monitoring was non-inferior to the other two groups [226]. Furthermore, information was published in environment maintenance guide for at-home respiration remote monitoring (draft) [227]. According to the research project report [228], CPAP remote monitoring addition is described as follows:

**c. Actual operation of the CPAP remote monitoring addition in Japan**

(1) Remote monitoring

- The vendor provides a usage report to the medical institution every month.
- Provided on paper or a USB
- Provided online using a cloud system

Either of the above is acceptable.

The physician-in-charge or a healthcare professional who receives instructions from the physician-in-charge checks the usage report every month. Based on the usage report, he/she will make sure to describe in the medical record the “number of days used,” “number of days used for 4 h or more, frequency,” “average usage time during use,” and “AHI,” among others, per month.

(2) Remote patient guidance or management

The physician in charge provides remote patient guidance or management to the patient himself/herself during the months when there is no face-to-face medical care. However, if the usage data for 1 month satisfies any of the following, it is possible to omit the guidance only for that month at the discretion of the attending physician.

- Good adherence: use for 4 h or more on 70% or more of treatment days.
- There is no significant deterioration in adherence: there is no decrease of 50% or more in any of “days used,” “days used for 4 h or more,” or “average hours used.”
- Good therapeutic effect: AHI less than 20.
- Remote patient guidance may not be instituted at the discretion of the attending physician.

Contact methods for remote patient guidance or management include email and fax, among others, but guidance is provided by telephone. When using the telephone, contact during normal consultation hours is the general rule. However, if the patient fails to get in touch after two telephone calls, there is no need to make any further telephone calls or other communication.

The content of remote patient guidance or management must be described in the medical record every month. In particular, this should include:

- Presence or absence of remote patient guidance
- Contact method used and contents
- Details, if no contact is made: e.g., contacted by phone on 10/20 and 10/22, but could not connect.

The above descriptions are essential. In the absence of any descriptions, it is assumed that remote patient guidance or management was not performed. Even if the guidance was omitted, make sure to describe it.

Remote patient guidance or management is limited to the content on adherence (“days of use,” “days of use for 4 hours or more,” “average usage time during use,” “AHI”, etc.). If problems such as treatment effect or mask fitting (leakage) are found in the usage report, face-to-face medical care should be provided.

## Publication bias

The “Sleep Apnea Syndrome (SAS) Clinical Practice Guideline 2020” was prepared to serve a dual purpose of disseminating new technologies and introducing evidence of current efficacy, the contents of which are based on scientific evidence and not influenced by any interests of any particular organization or product/technology. Furthermore, all the costs required to prepare this guideline were paid for by the Japanese Respiratory Society, with no support received from other organizations or companies.

Efforts were made to prepare unbiased recommendations based on the declared COI. Specifically, taking into consideration the COI of each member at the time of voting, if there was a COI in a voting item, the relevant member did not participate in the voting on said item.
